# Modelling Individual Differences in the Form of Pavlovian Conditioned Approach Responses: A Dual Learning Systems Approach with Factored Representations

**DOI:** 10.1371/journal.pcbi.1003466

**Published:** 2014-02-13

**Authors:** Florian Lesaint, Olivier Sigaud, Shelly B. Flagel, Terry E. Robinson, Mehdi Khamassi

**Affiliations:** 1 Institut des Systèmes Intelligents et de Robotique, UMR 7222, UPMC Univ Paris 06, Paris, France; 2 Institut des Systèmes Intelligents et de Robotique, UMR 7222, CNRS, Paris, France; 3 Department of Psychiatry, University of Michigan, Ann Arbor, Michigan, United States of America; 4 Molecular and Behavioral Neuroscience Institute, University of Michigan, Ann Arbor, Michigan, United States of America; 5 Department of Psychology, University of Michigan, Ann Arbor, Michigan, United States of America; Indiana University, United States of America

## Abstract

Reinforcement Learning has greatly influenced models of conditioning, providing powerful explanations of acquired behaviour and underlying physiological observations. However, in recent autoshaping experiments in rats, variation in the form of Pavlovian conditioned responses (CRs) and associated dopamine activity, have questioned the classical hypothesis that phasic dopamine activity corresponds to a reward prediction error-like signal arising from a classical Model-Free system, necessary for Pavlovian conditioning. Over the course of Pavlovian conditioning using food as the unconditioned stimulus (US), some rats (sign-trackers) come to approach and engage the conditioned stimulus (CS) itself – a lever – more and more avidly, whereas other rats (goal-trackers) learn to approach the location of food delivery upon CS presentation. Importantly, although both sign-trackers and goal-trackers learn the CS-US association equally well, only in sign-trackers does phasic dopamine activity show classical reward prediction error-like bursts. Furthermore, neither the acquisition nor the expression of a goal-tracking CR is dopamine-dependent. Here we present a computational model that can account for such individual variations. We show that a combination of a Model-Based system and a revised Model-Free system can account for the development of distinct CRs in rats. Moreover, we show that revising a classical Model-Free system to individually process stimuli by using factored representations can explain why classical dopaminergic patterns may be observed for some rats and not for others depending on the CR they develop. In addition, the model can account for other behavioural and pharmacological results obtained using the same, or similar, autoshaping procedures. Finally, the model makes it possible to draw a set of experimental predictions that may be verified in a modified experimental protocol. We suggest that further investigation of factored representations in computational neuroscience studies may be useful.

## Author Summary

Acquisition of responses towards full predictors of rewards, namely Pavlovian conditioning, has long been explained using the reinforcement learning theory. This theory formalizes learning processes that, by attributing values to situations and actions, makes it possible to direct behaviours towards rewarding objectives. Interestingly, the implied mechanisms rely on a reinforcement signal that parallels the activity of dopamine neurons in such experiments. However, recent studies challenged the classical view of explaining Pavlovian conditioning with a single process. When presented with a lever whose retraction preceded the delivery of food, some rats started to chew and bite the food magazine whereas others chew and bite the lever, even if no interactions were necessary to get the food. These differences were also visible in brain activity and when tested with drugs, suggesting the coexistence of multiple systems. We present a computational model that extends the classical theory to account for these data. Interestingly, we can draw predictions from this model that may be experimentally verified. Inspired by mechanisms used to model instrumental behaviours, where actions are required to get rewards, and advanced Pavlovian behaviours (such as overexpectation, negative patterning), it offers an entry point to start modelling the strong interactions observed between them.

## Introduction

Standard Reinforcement Learning (RL) [1] is a widely used normative framework for modelling conditioning experiments [2], [3]. Different RL systems, mainly Model-Based and Model-Free systems, have often been combined to better account for a variety of observations suggesting that multiple valuation processes coexist in the brain [4]–[6]. Model-Based systems employ an explicit model of consequences of actions, making it possible to evaluate situations by forward inference. Such systems best explain goal-directed behaviours and rapid adaptation to novel or changing environments [7]–[9]. In contrast, Model-Free systems do not rely on internal models and directly associate values to actions or states by experience such that higher valued situations are favoured. Such systems best explain habits and persistent behaviours [9]–[11]. Of significant interest, learning in Model-Free systems relies on a computed reinforcement signal, the reward prediction error (RPE). This signal parallels the observed shift of dopamine neurons' response from the time of an initially unexpected reward – an outcome that is better or worse than expected – to the time of the conditioned stimulus that precedes it, which, in Pavlovian conditioning experiments, is fully predictive of the reward [12], [13].

However recent work by Flagel et al. [14], raises questions about the exclusive use of classical RL Model-Free methods to account for data in Pavlovian conditioning experiments. Using an autoshaping procedure, a lever-CS was presented for 8 seconds, followed immediately by delivery of a food pellet into an adjacent food magazine. With training, some rats (sign-trackers; STs) learned to rapidly approach and engage the lever-CS. However, others (goal-trackers; GTs) learned to approach the food magazine upon CS presentation, and made anticipatory head entries into it. Furthermore, in STs, phasic dopamine release in the nucleus accumbens, measured with fast scan cyclic voltammetry, matched RPE signalling, and dopamine was necessary for the acquisition of a sign-tracking CR. In contrast, despite the fact that GTs acquired a Pavlovian conditioned approach response, this was not accompanied with the expected RPE-like dopamine signal, nor was the acquisition of a goal-tracking CR blocked by administration of a dopamine antagonist (see also [15]).

Classical dual systems models [16]–[19] should be able to account for these behavioural and pharmacological data, but the physiological data are not consistent with the classical view of RPE-like dopamine bursts. Based on the observation that STs and GTs focus on different stimuli in the environment, we suggest that the differences observed in dopamine recordings may be due to an independent valuation of each stimulus. In classical RL, valuation is usually done at the *state* level. Stimuli, embedded into *states* – snapshots of specific configurations in time –, are therefore hidden to systems. In this case, it would prevent dealing separately with the lever and the magazine at the same time. However, such data may still be explained by a dual systems theory, when extended to support and benefit from factored representations; that is, learning the specific value of stimuli independently from the states in which they are presented.

In this paper, we present and test a model using a large set of behavioural, physiological and pharmacological data obtained from studies on individual variation in Pavlovian conditioned approach behaviour [14], [20]–[25]. It combines Model-Free and Model-Based systems that provide the specific components of the observed behaviours [26]. It explains why inactivating dopamine in the core of the nucleus accumbens or in the entire brain results in blocking specific components and not others [14], [25]. By weighting the contribution of each system, it also accounts for the full spectrum of observed behaviours ranging from one extreme – sign-tracking – to the other [26] – goal-tracking. Above all, by extending classical Model-Free methods with factored representations, it potentially explains why the lever-CS and the food magazine might acquire different motivational values in different individuals, even when they are trained in the same task [22]. It may also account for why the RPE-like dopaminergic responses are observed in STs but not GTs, and also the differential dependence on dopamine [14].

## Results

**Figure 1:**
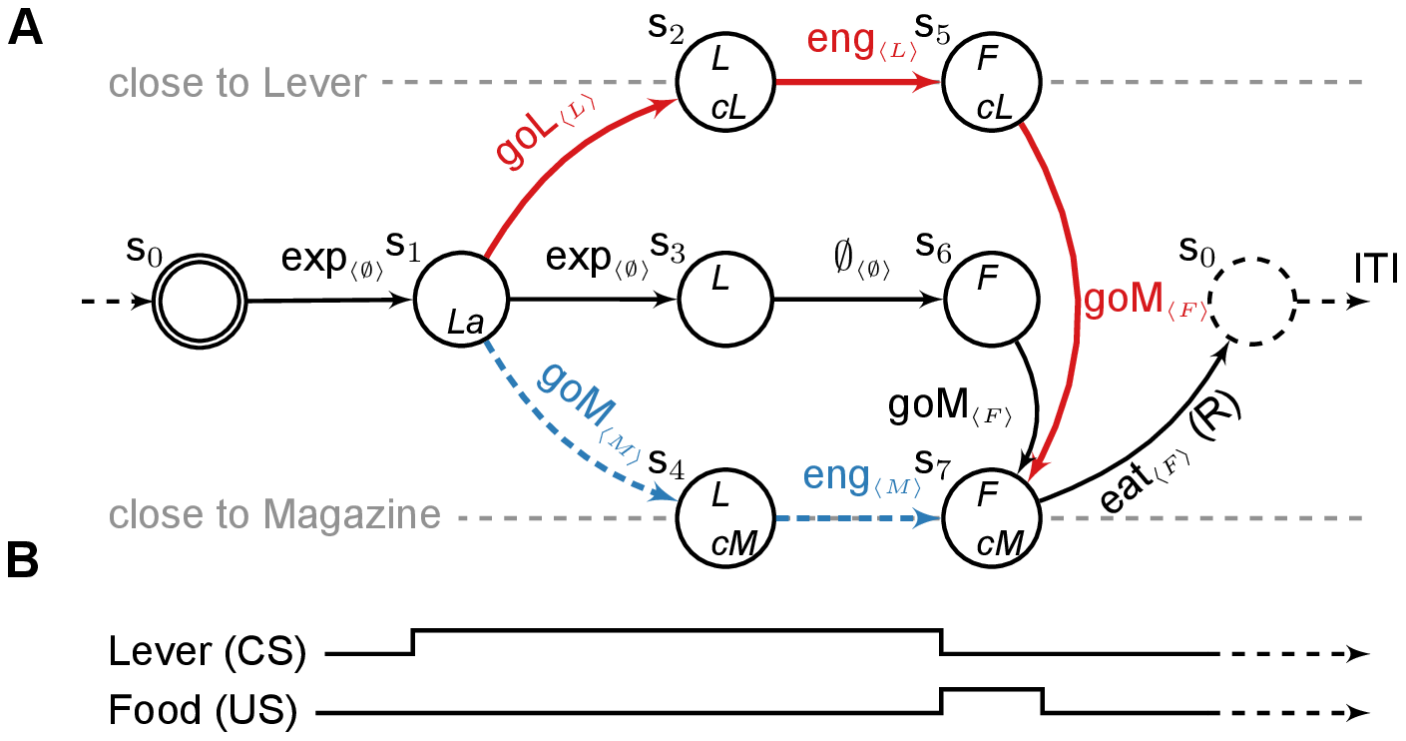
Computational representation of the autoshaping procedure. (**A**) MDP accounting for the experiments described in [14], [21], [22], [26]. States are described by a set of variables: *L*/*F* - Lever/Food is available, *cM*/*cL* - close to the Magazine/Lever, *La* - Lever appearance. The initial state is double circled, the dashed state is terminal and ends the current episode. Actions are *eng*age with the proximal stimuli, *exp*lore, or *go* to the *M*agazine/*L*ever and *eat*. For each action, the feature that is being focused on is displayed within brackets. The path that STs should favour is in red. The path that GTs should favour is in dashed blue. (**B**) Time line corresponding to the unfolding of the MDP.

We model the task as a simple Markov Decision Process (MDP) with different paths that parallel the diverse observed behaviours ranging from sign-tracking – engaging with the lever as soon as it appears – to goal-tracking – engaging with the magazine as soon as the lever-CS appears – (see Figure 1).

**Figure 2:**
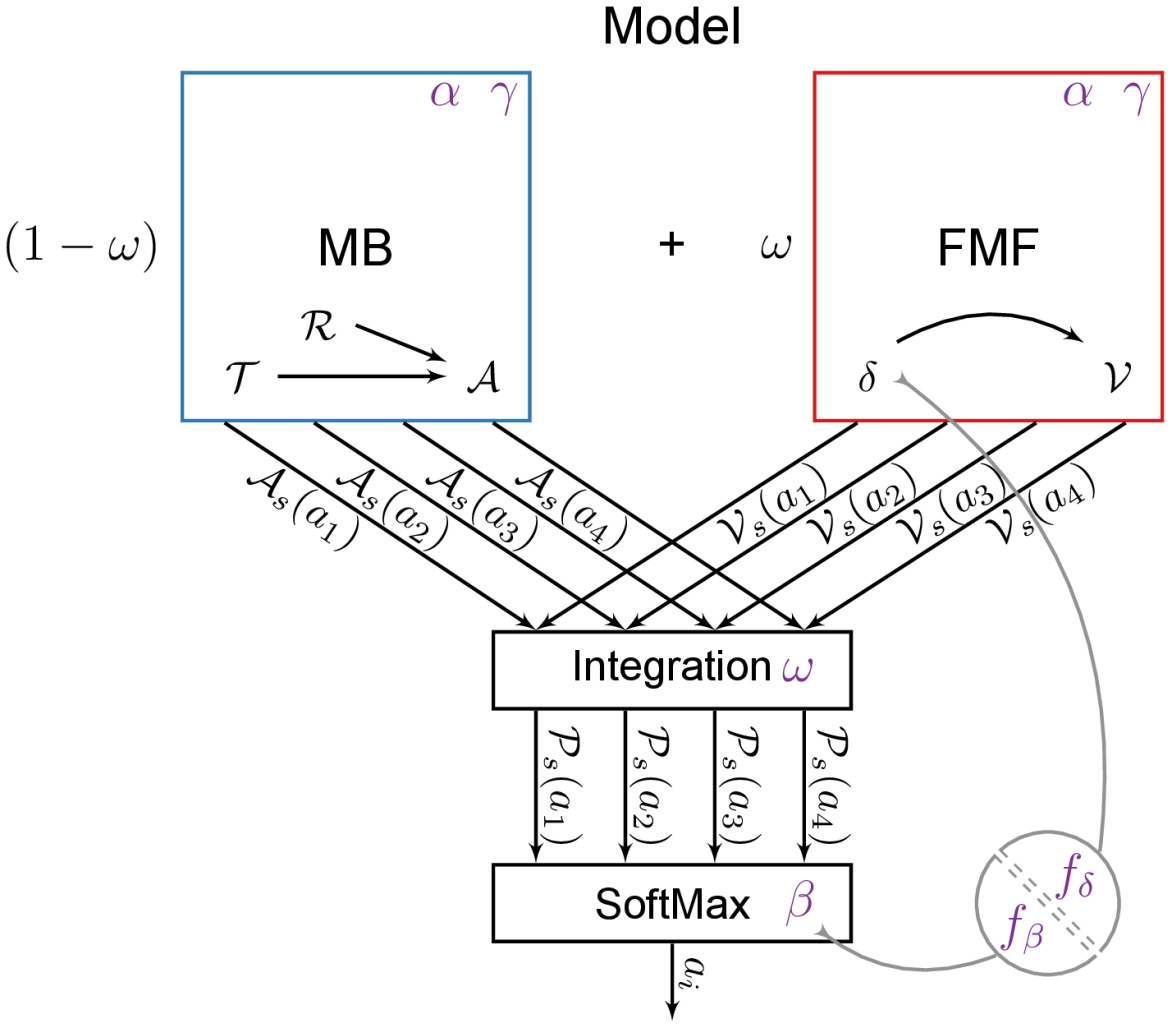
General architecture of the model and variants. The model is composed of a Model-Based system (MB, in blue) and a Feature-Model-Free system (FMF, in red) which provide respectively an Advantage function 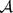 and a value function 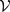 values for actions 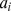 given a state 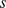. These values are integrated in 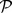, prior to be used into an action selection mechanism. The various elements may rely on parameters (in purple). The impact of flupentixol on dopamine is represented by a parameter 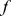 that influences the action selection mechanism and/or any reward prediction error that might be computed in the model.

The computational model (see Figure 2) consists of two learning systems, employing distinct mechanisms to learn the same task: (1) a Model-Based system which learns the structure of the task from which it infers its values; (2) a Feature-Model-Free system where values for the relevant stimuli (lever-CS and the food magazine) are directly learned by trial and error using RPEs. The respective values of each system are then weighted by an 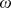 parameter before being used in a classical softmax action-selection mechanism (see Methods).

An important feature of the model is that varying the systems weighting parameter 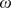 (while sharing the other parameter values of the model across subgroups) is sufficient to qualitatively reproduce the characteristics of the different subgroups of rats observed experimentally during these studies.

To improve the matching of the following results with the main experimental data, a different set of parameter values was used for each subgroup (ST, GT and IG). The values were retrieved after fitting autoshaping data only (see Methods, Table S1). Simulated results on other behavioural, physiological and pharmacological data are generated with the same parameter values. While it might result in a weaker fitting of the other experimental data, this permits a straightforward comparison of results at different levels for the same simulation. Moreover, it confirms that the model can reproduce behavioural, physiological and pharmacological results with a single simulation per subgroup.

**Figure 3:**
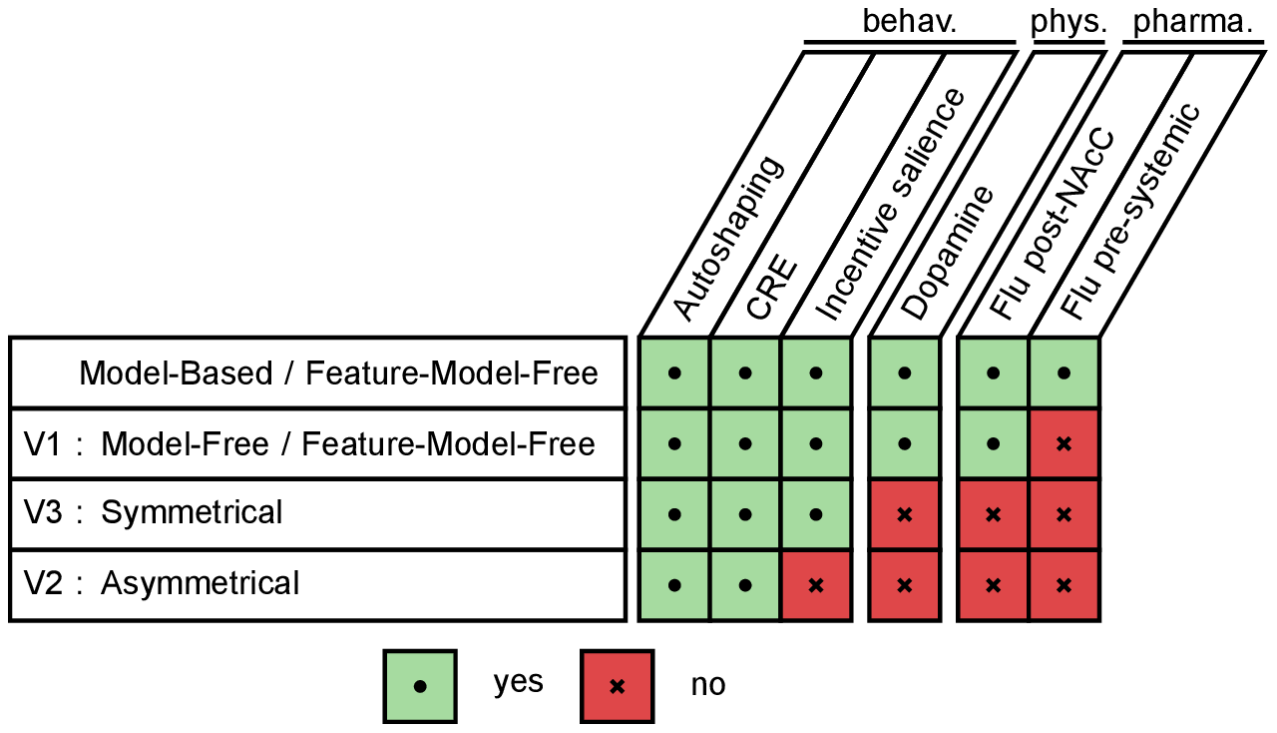
Summary of simulations and results. Each line represents a different model composed of a pair of Reinforcement Learning systems. Each column represents a simulated experiment. Experiments are grouped by the kind of data accounted for: behavioural (autoshaping [14], [21], CRE [22], Incentive salience [23], [24]), physiological [21] and pharmacological (Flu post-NAcC [25], Flu pre-systemic [21]). Variant 4 (i.e. Model-based/Model-Free without features) is not included as it failed to even reproduce the autoshaping behavioural results and was not investigated further.

**Figure 4:**
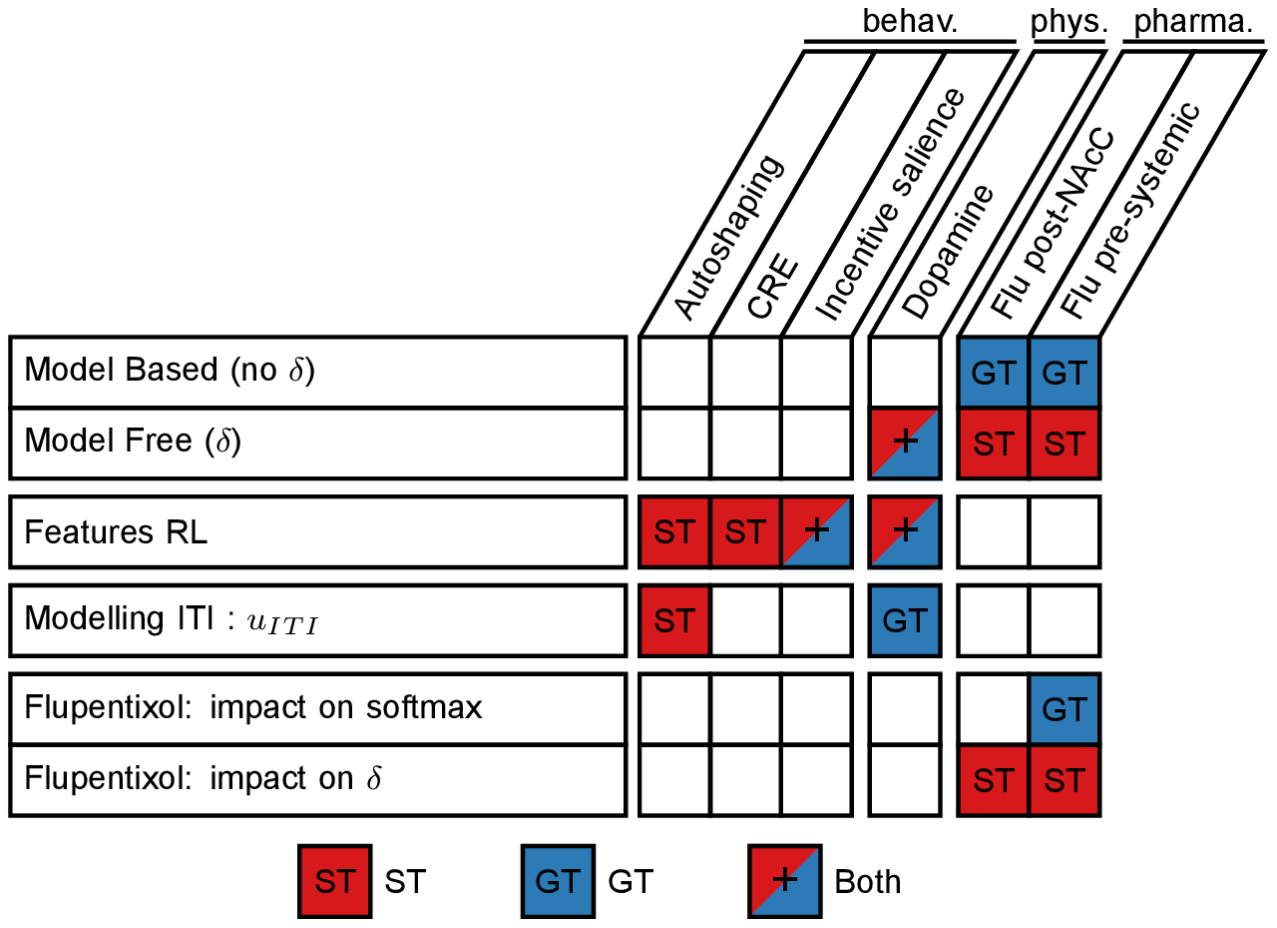
Summary of the key mechanisms required by the model to reproduce experimental results. Each line represents a different mechanism of the model. Each column represents a simulated experiment. For each mechanism, it states in which experiment and for which behaviour – sign-tracking (red), goal-tracking (blue) or both (+) – it is required. Note however that all mechanisms and associated parameters have, to a certain extent, an impact on any presented results.

On each set of experimental data, we compare different variants of the computational model in order to highlight the key mechanisms that are required for their reproduction. Simulation results on each data subset are summarized in Figure 3. The role of each specific mechanism of the model in reproducing each experimental data is detailed in Figure 4.

### Behavioural data

#### Autoshaping

The central phenomenon that the model is meant to account for is the existence of individual behavioural differences in the acquisition of conditioned approach responses in rats undergoing an autoshaping procedure; that is, the development of a sign-tracking CR, a goal-tracking CR, or an intermediate response.

**Figure 5:**
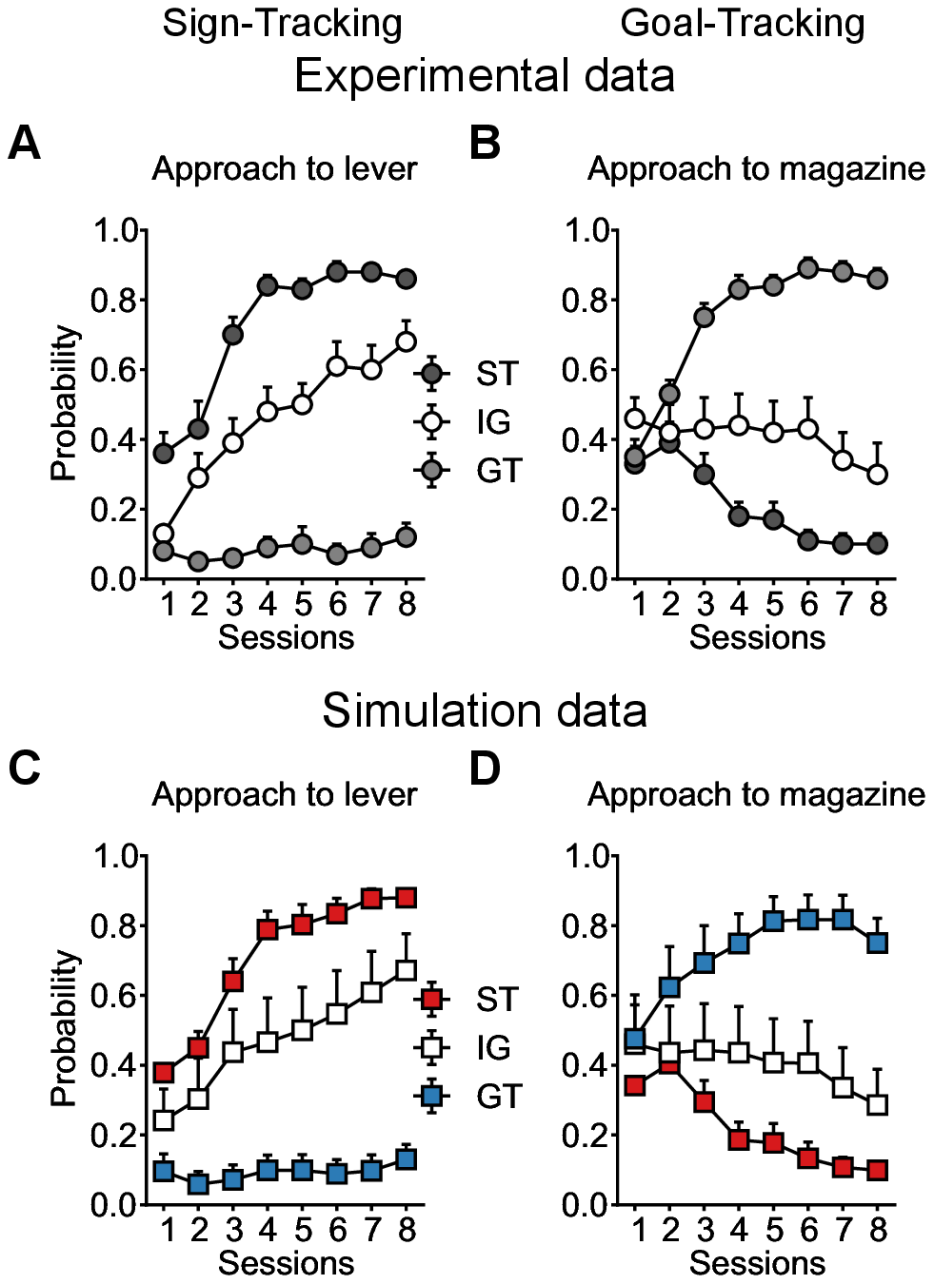
Reproduction of sign- versus goal-tracking tendencies in a population of rats undergoing an autoshaping experiment. Mean probabilities to engage at least once with the lever (**A,C**) or the magazine (**B,D**) during trials. Data are expressed as mean 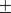 S.E.M. and illustrated in 50-trial (2-session) blocks. (**A,B**) Reproduction of Flagel et al. [21] experimental results (Figure 2 A,B). Sign-trackers (ST) made the most lever presses (black), goal-trackers (GT) made the least lever presses (white), Intermediate group (IG) is in between (grey). (**C,D**) Simulation of the same procedure (squares) with the model. Simulated groups of rats are defined as STs (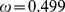; 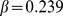; 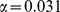; 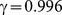; 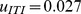; 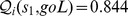; 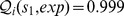; 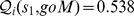; n = 14) in red, GTs (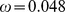 ; 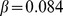; 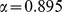; 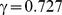; 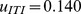; 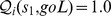; 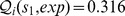; 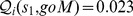; n = 14) in blue and IGs (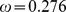; 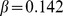; 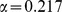; 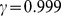; 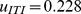; 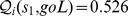; 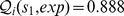; 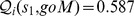; n = 14) in white. The model reproduces the same behavioural tendencies. With training, STs tend to engage more and more with the lever and less with the magazine, while GTs neglect the lever to increasingly engage with the magazine. IGs are in between.

Based on their engagement towards the lever, Flagel et al. [21] divided rats into three groups (see [26] for a more recently defined criterion). At lever appearance, rats that significantly increased their engagement towards it (top 30%) were classified as STs, whereas rats that almost never engaged with the lever (bottom 30%) were classified as GTs (these latter animals engaged the food magazine upon CS presentation). The remaining rats, engaging in both lever and magazine approach behaviours were defined as the Intermediate Group (IGs) (see Figure 5 A, B). STs and GTs acquired their respective CRs at a similar rate over days of training [22].

The current model is able to reproduce such results (see Figure 5 C, D). By running a simulation for each group of rats, using different parameters (mainly varying the 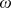 parameter) the model reproduces the different tendencies to engage with the lever (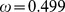), with the magazine (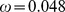) or to fluctuate between the two (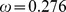). A high 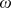 strengthens the influence of the Feature-Model-Free system, which learns to associate a high motivational value to the lever CS, and a sign-tracking CR dominates. A low 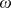 increases the influence of the Model-Based system, which infers the optimal behaviour to maximize reward, and goal-tracking is favoured. When both systems are mixed, i.e. with an intermediate 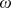, the behaviour is more likely to oscillate between sign- and goal-tracking, representative of the intermediate group.

These results rely on the combination of two systems that would independently lead to ‘pure’ sign-tracking or goal-tracking CRs. Three tested variants of the model could reproduce these behavioural results as well (see Figure S1): a combination of Feature-Model-Free systems and simple Model-Free system (Variant 1); a multi-step extension of Dayan 2006's model [16] giving a Pavlovian impetus for the lever (Variant 2); and a symmetrical version of this last model with two impetuses, one for the lever, and one for the magazine (Variant 3) (see Methods). Interestingly, a combination of Model-Based and classical Model-Free (not feature-based : Variant 4) fails in reproducing these results (see Figure S8). This is because both systems are proven to converge to the same values and both would favour pure goal-tracking, such that varying their contribution has no impact on the produced behaviours.

Thus, at this stage, we can conclude that several computational models based on dual learning systems can reproduce these behavioural results, given that the systems favour different behaviours (see Figure S1). However, Variants 1, 2 and 3 fail to reproduce other behavioural, pharmacological and physiological data characteristic of STs and GTs (see following sections).

#### Incentive salience

**Figure 6:**
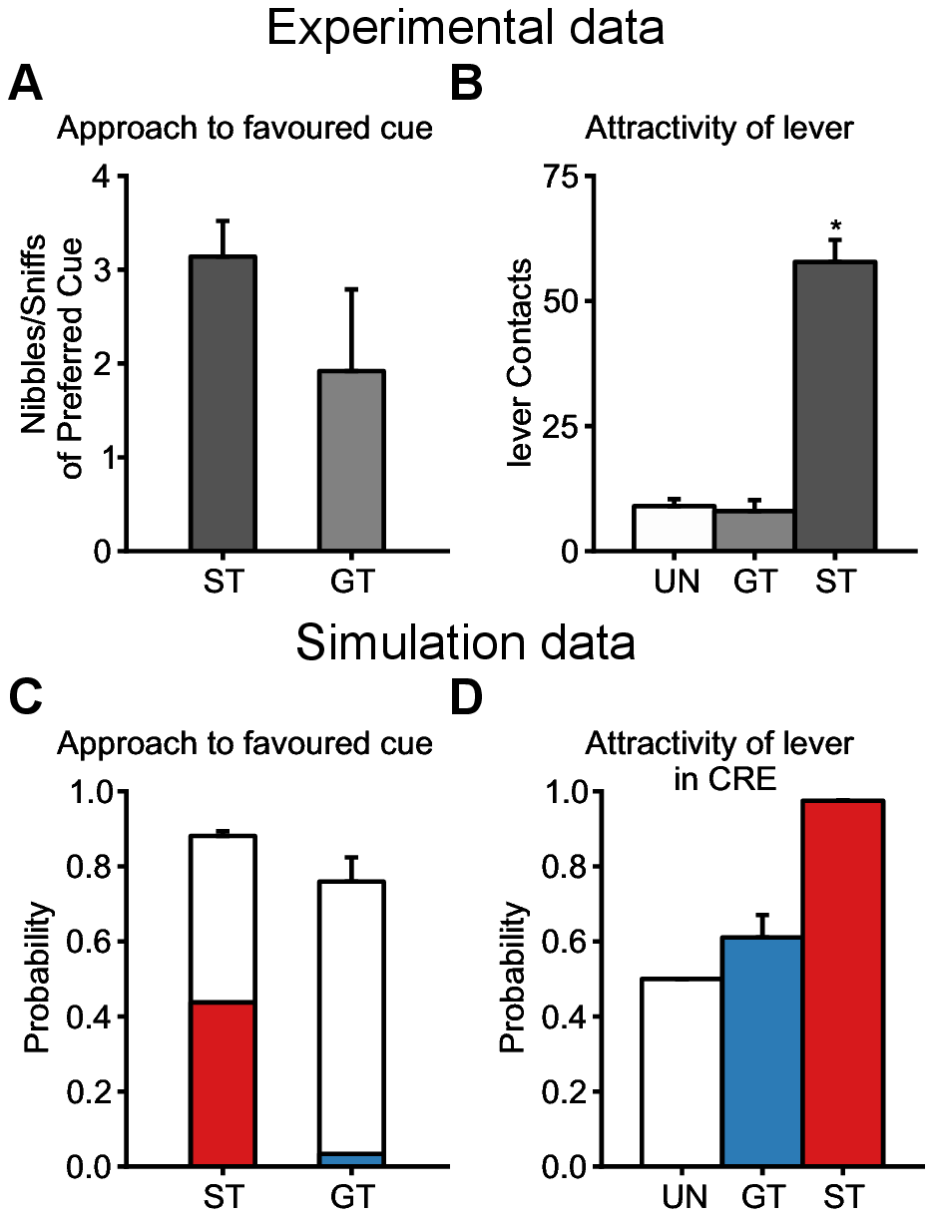
Possible explanation of incentive salience and Conditioned Reinforcement Effect by values learned during autoshaping procedure. Data are expressed as mean 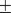 S.E.M. Simulated groups of rats are defined as in Figure 5. (**A**) Number of nibbles and sniffs of preferred cue by STs and GTs as a measure for incentive salience. Data extracted from Mahler et al. [23] from Figure 3 (bottom-left). (**B**) Reproduction of Robinson et al. [22] experimental results (Figure 2 B). Lever contacts by STs and GTs during a conditioned reinforcer experiment. (**C**) Probability to engage with the respective favoured stimuli of STs and GTs at the end of the simulation (white, similar to the last session of Figure 5 C for STs and D for GTs) superimposed with the contribution in percentage of the values attributed by the Feature-Model-Free system in such engagement for STs (red) and GTs (blue). We hypothesize that such value is the source of incentive salience and explains why STs and GTs have a consumption-like behaviour towards their favoured stimulus. (**D**) Probability to engage with the lever versus exploring when presented with the lever and no magazine for STs (red), GTs (blue) and a random-policy group UN (white), simulating the unpaired group (UN) of the experimental data. Probabilities were computed by applying the softmax function after removing the values for the magazine interactions (see Methods). STs would hence actively seek to engage with the lever relatively to GTs in a Conditioned Reinforcement Effect procedure.

The results in Figure 5 only represent the probability of approach to either the lever-CS or the food magazine. Thus, they do not account for the specific ways rats engage and interact with the respective stimuli. In fact, if food is used as the US, rats are known to chew and bite the stimuli on which they are focusing [23], [24] (see Figure 6 A). Importantly, both STs and GTs express this consumption-like behaviour during the CS period, directed towards the lever or the food magazine, respectively. It has been argued that this behaviour may reflect the degree to which incentive salience is attributed to these stimuli, and thus the extent to which they become “wanted” [23], [24], [27].

In an RL-like framework, incentive salience attribution can be represented as a bonus mechanism for interacting with stimuli. The Feature-Model-Free system in the model realizes such a function, providing a specific bonus for each stimulus in any simulated rat. Such bonus was inspired by the Pavlovian impetus mechanism of Dayan 2006's model [16]. Figure 6 C shows the percentage of Feature-Model-Free value that contributed to the computation of the probability to engage with the respective favoured cues of STs and GTs at the end of the simulation.

The presence of the magazine in the inter-trial interval (ITI), and the necessary revision of the associated bonus at a lower value when exploring, makes the associated bonus smaller than that of the lever (see Methods). This results in a even smaller contribution of this bonus in GTs behaviour (blue bar in Figure 6 C) compared to STs (red bar in Figure 6 C). Although it is not straightforward to interpret how the probability of engagement (white bars in Figure 6 C) in the model might be translated into a consumption-like behaviour from a computational point of view, we propose that the different contributions of bonuses could explain the slightly smaller number of nibbles and sniffs of preferred cue observed experimentally in GTs compared to STs (Figure 6 A, adapted from [23]). This may also explain why other studies have observed a smaller proportion of nibbles on the magazine in GTs [24] and less impulsiveness [28] in GTs compared to STs. We come back to this issue in the discussion.

Variants 1 and 3 also realize such function by providing bonuses for actions leading to both stimuli (see Figure S2). Only providing bonus for sign-tracking behaviour – as in Dayan's model (Variant 2) – does not fit well with the attribution of incentive salience to both stimuli. It would suggest that we should not observe incentive salience towards the magazine in any rats, which is in discrepancy with the experimental data. Thus, the important mechanism here is that stimuli are not processed differently. Any stimulus is attributed with its respective bonus, which is pertinent in regard to the attribution of incentive salience.

#### Conditioned Reinforcement Effect (CRE)

An important question about the difference in observed behaviours is about the properties acquired by the lever that makes it more attractive to STs than to GTs. To answer this question, Robinson and Flagel studied the dissociation of the predictive and motivational properties of the lever [22]. Part of their results involves asking whether the Pavlovian lever-CS would serve as a conditioned reinforcer, capable of reinforcing the learning of a new instrumental response [29], [30]. In a new context, rats were presented with an active and an inactive nose port. Nose poking into the active port resulted in presentation of the lever for 2 seconds without subsequent reward delivery, whereas poking into the inactive one had no consequence. The authors observed that while both STs and GTs preferred the active nose port to an inactive one, STs made significantly more active nose pokes than GTs (see Figure 6 B, see also [31]). This suggests that the lever acquired greater motivational value in STs than in GTs.

Without requiring additional simulations, the model can explain these results by the value that has been incrementally learned and associated with approaching the lever in the prior autoshaping procedure for STs and GTs. In the model, STs attribute a higher value to interacting with the lever than GTs and should actively work for its appearance enabling further engagement. Figure 6 D shows the probabilities of engagement that would be computed at lever appearance after removing the magazine (and related actions) at the end of the experiment. Indeed, even though the lever is presented only very briefly, upon its presentation in the conditioned reinforcement test, STs actively engage and interact with it [22]. Any value associated to a state-action pair makes this action in the given state rewarding in itself, favouring actions (e.g. nosepokes) that would lead to such state. Repeatedly taking this action without receiving rewards should eventually lead to a decrease of this value and reduce the original engagement.

### Physiological data

Not only have Flagel et al. [14] provided behavioural data but they also provide physiological and pharmacological data. This raises the opportunity to challenge the model at different levels, as developed in the current and next sections.

**Figure 7:**
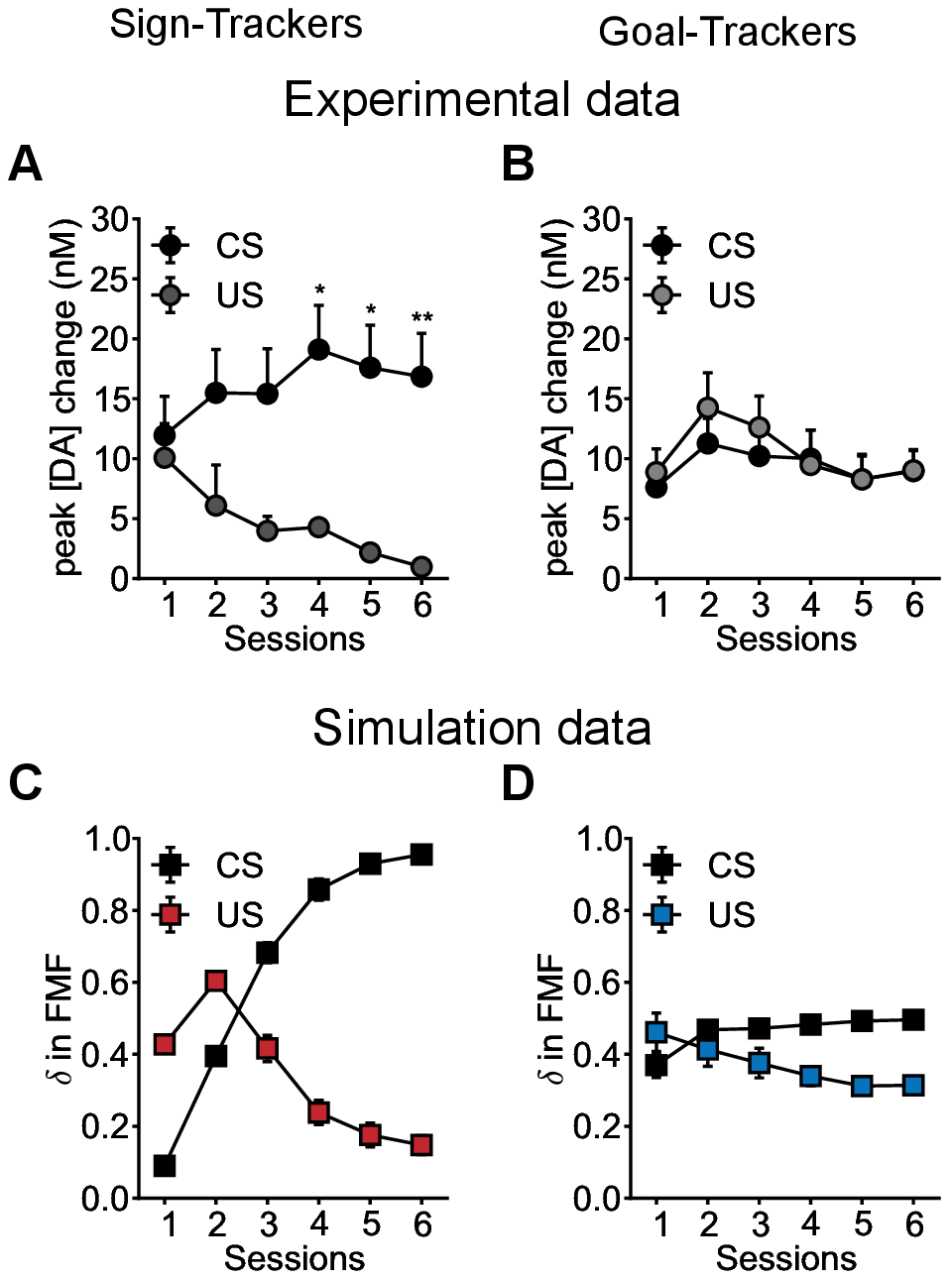
Reproduction of patterns of dopaminergic activity of sign- versus goal-trackers undergoing an autoshaping experiment. Data are expressed as mean 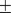 S.E.M. (**A,B**) Reproduction of Flagel et al. [14] experimental results (Figure 3 d,f). Phasic dopamine release recorded in the core of the nucleus accumbens in STs (light grey) and GTs (grey) using Fast Scan Cyclic Voltammetry. Change in peak amplitude of the dopamine signal observed in response to CS and US presentation for each session of conditioning (**C,D**) Average RPE computed by the Feature-Model-Free system in response to CS and US presentation for each session of conditioning. Simulated groups of rats are defined as in Figure 5. The model is able to qualitatively reproduce the physiological data. STs (blue) show a shift of activity from US to CS time over training, while GTs develop a second activity at CS time while maintaining the initial activity at US time.

Using Fast Scan Cyclic Voltammetry (FSCV) in the core of the nucleus accumbens they recorded the mean of phasic dopamine (DA) signals upon CS (lever) and US (food) presentation. It was observed that depending on the subgroup of rats, distinct dopamine release patterns emerge (see Figure 7 A,B) during Pavlovian training. STs display the classical propagation of a phasic dopamine burst from the US to the CS over days of training and the acquisition of conditioned responding (see Figure 7 A). This pattern of dopamine activity is similar to that seen in the firing of presumed dopamine cells in monkeys reported by Schultz and colleagues [12] and interpreted as an RPE corresponding to the reinforcement signal 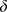 of Model-Free RL systems [1]. In GTs, however, a different pattern was observed. Initially there were small responses to both the CS and US, of which the amplitudes seemed to follow a similar trend over training (see Figure 7 B).

By recording the mean of the RPEs 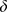 computed in the Feature-Model-Free system during the autoshaping simulation (i.e. only fitted to behavioural data), the model can still qualitatively reproduce the different patterns observed in dopamine recordings for STs and GTs (see Figure 7 C,D). For STs, the model reproduces the progressive propagation of 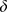 from the US to the CS (see Figure 7 C). For GTs, it reproduces the absence of such propagation. The RPE at the time of the US remains over training, while a 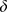 also appears at the time of the CS (see Figure 7 D). In the model, such discrepancy is explained by the difference in the values that STs and GTs use for the computation of RPEs at the time of the CS and the US. STs, by repeatedly focusing on the lever, propagate the total value of food to the lever and end up having a unique 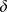 at the unexpected lever appearance only. By contrast, by repeatedly focusing on the magazine during the lever appearance but, as all rats, also from time to time during ITI, GTs revise the magazine value multiple times, positively just after food delivery and negatively during ITI. Such revisions lead to a permanent discrepancy between the expected and observed value, i.e. a permanent 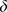, at lever appearance and food delivery, when engaging with the magazine.

The key mechanism to reproduce these results resides in the generalization capacities of the Feature-Model-Free system. Based on features rather than states, feature-values are to be used, and therefore revised, at different times and states of the experiment, favouring the appearance of RPEs. Variants 2, 3 and 4 relying on classical Model-Free systems are unable to reproduce such results (see Figure S3). By using values over abstract states rather than stimuli, it makes it impossible to only revise the value of the magazine during ITI. Therefore, given the deterministic nature of the MDP, we observe a classical propagation of RPEs in all pathways up to the appearance of the lever.

### Pharmacological data

#### Effects of systemic flupentixol administration on the learning of sign- and goal-tracking behaviours

Flagel et al. [14] also studied the impact of systemic injections of the non specific dopamine antagonist, flupentixol, on the acquisition of sign-tracking and goal-tracking CRs. The authors injected flupentixol in rats prior to each of 7 sessions and observed the resulting behaviours. Behaviour during the 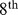 session was observed without flupentixol.

**Figure 8:**
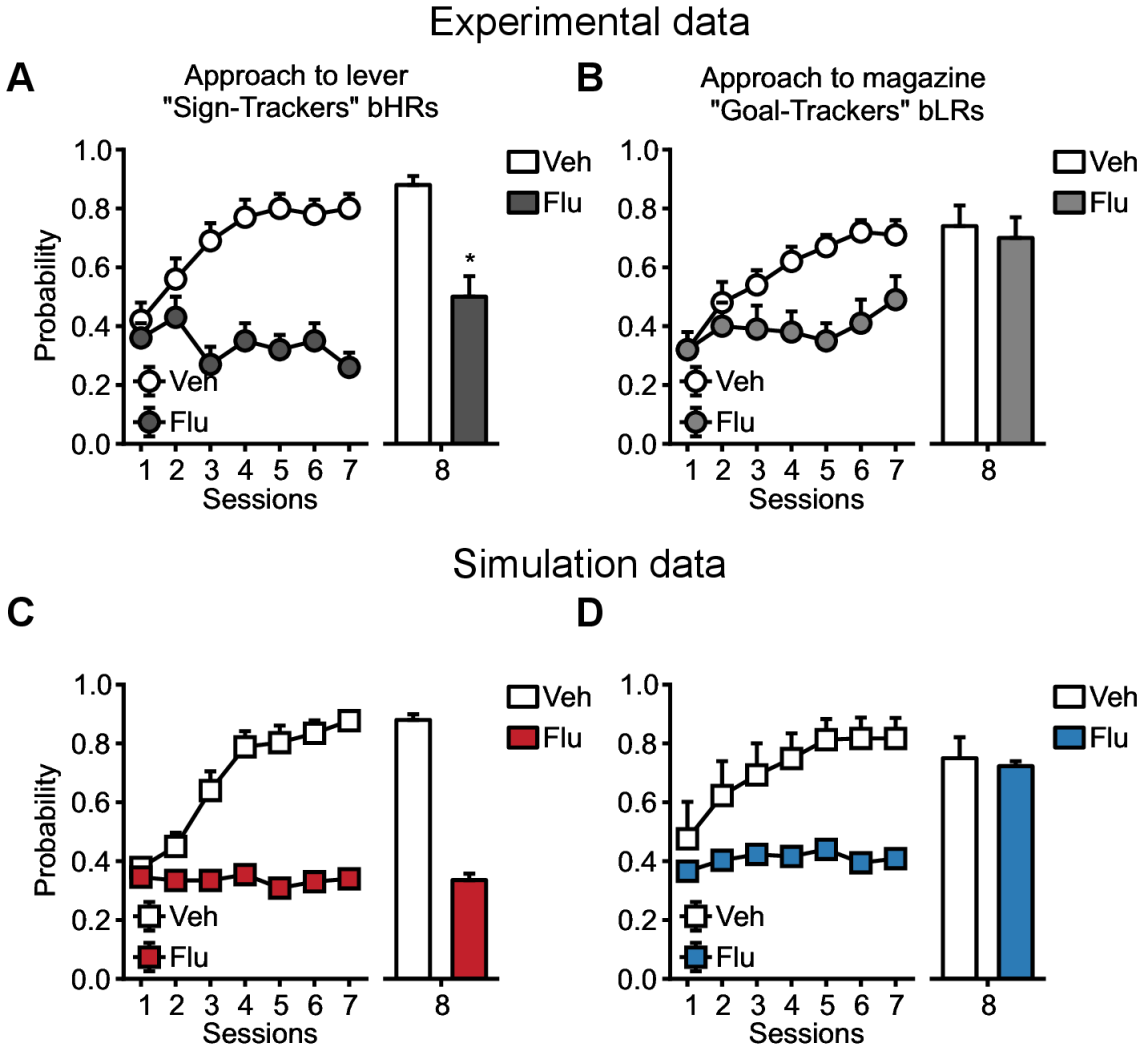
Reproduction of the effect of systemic injections of flupentixol on sign-tracking and goal-tracking behaviours. Data are expressed as mean 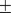 S.E.M. (**A,B**) Reproduction of Flagel et al. [14] experimental results (Figure 4 a,d). Effects of flupentixol on the probability to approach the lever for STs (**A**) and the magazine for GTs (**B**) during lever presentation. (**C,D**) Simulation of the same procedure (squares) with the model. Simulated groups of rats are defined as in Figure 5. (**C**) By flattening the softmax temperature and reducing the RPEs of the Feature-Model-Free system, to mimic the possible effect of flupentixol, the model can reproduce the blocked acquisition of sign-tracking in STs (red), engaging less the lever relatively to a saline-injected control group (white). (**D**) Similarly, the model reproduces that goal-tracking was learned but its expression was blocked. Under flupentixol (first 7 sessions), GTs (blue) did not express goal-tracking, but on a flupentixol-free control test (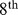 session) their engagement with the magazine was almost identical to the engagement of a saline-injected control group (white).

Systemic injections of flupentixol in STs and GTs (Flu groups, black curves in Figure 8 A,B) blocked expression of their respective behaviours during training. Saline injections (white curves in Figure 8 A,B) left their performances intact. The crucial test for learning took place on the 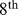 day, when all rats were tested without flupentixol. STs failed to approach the lever, and performed as the saline-injected controls did on the first day of training.

Thus, in STs flupentixol blocked the acquisition of a sign-tracking CR (see Figure 8 A). Interestingly, on the flupentixol-free test day GTs did not differ from the saline-injected control group, indicating that flupentixol did not block the acquisition of a goal-tracking CR (see Figure 8 B). Thus, acquisition of a sign-tracking CR, but not a goal-tracking CR, is dependent on dopamine (see also [15]).

The model reproduces these pharmacological results (see Figure 8 C,D). As in the experimental data, simulated GTs and STs do not show a specific conditioned response during the first 7 sessions under flupentixol. On the 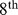 session, without flupentixol, we observe that STs still do not show a specific conditioned response while GTs perform at a level close to that of the saline-injected control group (see Figure 8 C,D).

The absence of specific conditioned response in the whole population for the first 7 sessions is first due to the hypothesized [32] impact of flupentixol on action selection (see Methods). With enough flupentixol, the elevation of the selection temperature leads to a decrease of the influence of learned values in the expressed behaviour, masking any possibly acquired behaviour.

The absence of a specific conditioned response in STs is due to the blockade of learning in the second system by flupentixol, since it is RPE-dependent. Therefore almost no learning occurs in the system (see Figure 8).

In contrast, with the first system being RPE-independent, flupentixol has no effect on learning, because it is Model-Based rather than Model-Free [33]. The expression of behaviour is blocked at the action selection level, which does not make use of values learned by the Model-Based system. Thus, GTs, relying mainly on the first system, learn their CR under flupentixol but are just not able to express it until flupentixol is removed. The lower level of goal-tracking in the Flu group relative to the saline-injected control group on the 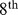 session is due to the lack of exploitation induced by flupentixol injection during the previous 7 sessions. By engaging less with the magazine, the Flu group ends up associating a lower value to the magazine (i.e. the value did not fully converge in 7 sessions) to guide its behaviour.

Interestingly, if the model had been constituted of Model-Free systems only – as in Variants 1, 2 and 3 – it would not have been able to reproduce these results, because both systems would have been RPE-dependent and thus sensitive to the effect of flupentixol (see Figure S4).

#### Effects of local flupentixol administration on the expression of sign- and goal-tracking behaviours

In a related experiment, Saunders et al. [25] studied the role of dopamine in the nucleus accumbens core in the expression of Pavlovian-conditioned responses that had already been acquired. After the same autoshaping procedure as in [20], they injected different doses of flupentixol in the core of the nucleus accumbens of rats and quantified its impact on the expression of sign-tracking and goal-tracking CRs in an overall population (without distinguishing between STs and GTs).

**Figure 9:**
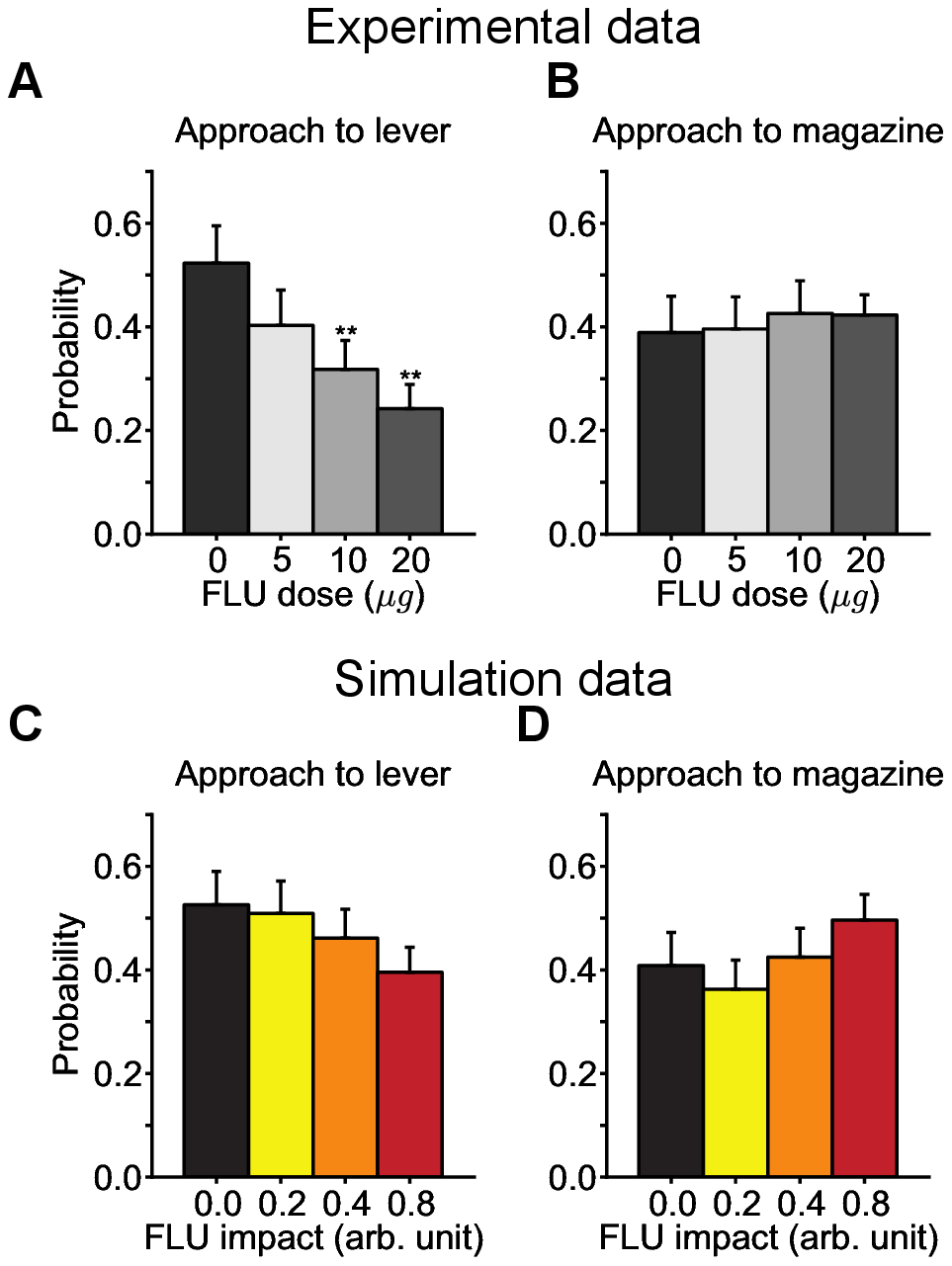
Reproduction of the effect of post injections of flupentixol in the core of the nucleus accumbens. Data are expressed as mean 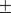 S.E.M. (**A,B**) Reproduction of Saunders et al. [25] experimental results (Figure 2 A,D). Effects of different doses of flupentixol on the general tendency to sign-track (**A**) and goal-track (**B**) in a population of rats, without discriminating between sign- and goal-trackers. (**C,D**) Simulation of the same procedure with the model. The simulated population is composed of groups of rats defined as in Figure 5. By simulating the effect of flupentixol as in Figure 8, the model is able to reproduce the decreasing tendency to sign-track in the overall population by increasing the dose of flupentixol.

They found that flupentixol dose dependently attenuated the expression of sign-tracking, while having essentially no effect on goal-tracking (see Figure 9 A, B). Along with the Flagel et al. [14] study, these results suggest that both the acquisition and expression of a sign-tracking CR is dopamine-dependent (at least in the core) whereas the acquisition and expression of a goal-tracking CR is not.

Given the assumption that the Feature-Model-Free system would take place in or rely on the core of the nucleus accumbens, this model reproduces the main experimental result: the decreased tendency to sign-track in the population (see Figure 9 C). Note that in the previous experiment, the injection of flupentixol was systemic, and assumed to affect any region of the brain relying on dopamine, whereas in the present experiment it was local to the core of the nucleus accumbens. Therefore, we modelled the impact of flupentixol differently between the current and previous simulations (see Methods). In the model, the tendency to sign-track is directly correlated with a second operational system. Any dysfunction in the learning process (here by a distortion of RPEs) reduces this trend.

The model successfully reproduced the absence of reduction of goal-tracking, in contrast to the reduction of sign-tracking. However, it was unable to reproduce the invariance in goal-tracking (see Figure 9 D) and rather produced an increase in goal-tracking. This is due to the use of a softmax operator for action selection, as this is the case in the vast majority of computational neuroscience RL models [16]–[19], [32], [34]–[36], which automatically favours goal-tracking when sign-tracking is blocked (see Limitations). We did not attempt to cope with this limitation because our focus here was the absence of reduction of goal-tracking.

Besides, the model could, after re-learning, reproduce the selective impact of intra-accumbal flupentixol injections observed in sign-tracking but not in goal-tracking, because such injections affected the learning process in the Feature-Model-Free system only.

## Discussion

We tested several mechanisms from the current literature on modelling individual variation in the form of Pavlovian conditioned responses (ST vs GT) that emerge using a classical autoshaping procedure, and the role of dopamine in both the acquisition and expression of these CRs. Benefiting from a rich set of data, we identified key mechanisms that are sufficient to account for specific properties of the observed behaviours. The resulting model relies on two major concepts: Dual learning systems and factored representations. Figure 4 summarizes the role of each mechanism in the model.

### Dual learning systems

Combining Model-Based and Model-Free systems has previously been successful in explaining the shift from goal-directed to habitual behaviours observed in instrumental conditioning [17]–[19], [33], [34]. However, few models based on the same concept have been developed to account for Pavlovian conditioning [16]. While the need for two systems is relevant in instrumental conditioning given the distinct temporal engagement of each system, such a distinction has not been applied to Pavlovian phenomena (but see recent studies on orbitofrontal cortex [37]–[39]). The variability of behaviours and the need for multiple systems have been masked by focusing on whole populations and, for the most part, ignoring individual differences in studies of Pavlovian conditioning. The nature of the CS is especially important, as many studies of Pavlovian conditioned approach behaviour have used an auditory stimulus as the CS, and in such cases only a goal-tracking CR emerges in rats [40], [41].

As expected from the behavioural data, combining two learning systems was successful in reproducing sign- and goal-tracking behaviours. The Model-Based system, learning the structure of the task, favours systematic approach towards the food magazine, and waiting for food to be delivered, and hence the development of a goal-tracking CR. The Feature-Model-Free system, directly evaluating features by trials and errors, favours systematic approach towards the lever, a full predictor of food delivery, and hence the development of a sign-tracking CR. Moreover, utilizing the Feature-Model-Free system to represent sign-tracking behaviour yields results consistent with the pharmacological data. Disrupting RPEs, which reflects the effects of flupentixol on dopamine, blocks the acquisition of a sign-tracking CR, but not a goal-tracking CR. The model does not make a distinction between simple approach behaviour versus consumption-like engagement, as reported for both STs and GTs [23], [24]. However given that such engagement results from the development of incentive salience [23], [24], the values learned by the Feature-Model-Free system to bias behaviour towards stimuli attributed with motivational value are well-suited to explain such observations. The higher motivational value attributed to the lever by STs relative to GTs can also explain why the lever-CS is a more effective conditioned reinforcer for STs than for GTs [22].

Importantly, none of the systems are dedicated to a specific behaviour, nor rely on *a priori* information to guide their processes. The underlying mechanisms increasingly make one behaviour more pronounced than the other through learning. Each system contributes to a certain extent to sign- and goal-tracking behaviour. This property is emphasized by the weighted sum integration of the values computed by each system before applying the softmax action-selection mechanism. The variability of behaviours in the population can then be accounted for by adjusting the weighting parameter 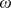 from 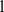 (i.e. favouring sign-tracking) to 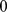 (i.e. favouring goal-tracking). This suggests that the rats' actions result from some combination of rational and impulsive processes, with individual variation contributing to the weight of each component.

The integration mechanism is directly inspired by the work of Dayan et al. [16] and as the authors suggest, the parameter 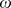 may fluctuate over time, making the contribution of the two systems vary with experience. In contrast to their model, however, the model presented here does not assign different goals to each system. Thus, the current model is more similar to their previous model [17], which uses another method for integration.

A common alternative to integration when using multiple systems [17], [18], [35] is to select at each step, based on a given criterion (certainty, speed/accuracy trade-off, energy cost), a single system to pick the next action. Such switch mechanism does not fit well with the present model, given that it would be interpreted as if actions relied sometimes only on motivational values (i.e. Feature-Model-Free system) and sometimes only on a rational analysis of the situation (i.e. Model-Based system). It also does not fit well with pharmacological observation that STs do not express goal-tracking tendencies in the drug-free test session following systemic-injections of flupentixol [14], as Flagel et al. stated, “[sign-tracking] rats treated with flupentixol did not develop a goal-tracking CR”.

### Factored representations

Classical RL algorithms used in neuroscience [16]–[18], [35], designed mainly to account for instrumental conditioning, work at the state level. Tasks are defined as graphs of states, and corresponding models are unaware of any similarity within states. Therefore, any subsequent valuation process cannot use any underlying structure to generalize updates to states that share stimuli. Revising the valuation process to handle features rather than states *per se*, makes it possible to attribute motivational values to stimuli independently of the states in which they are presented.

Recent models dedicated to Pavlovian conditioning [36], [42]–[46] usually represent and process stimuli independently and can be said to use factored representations, a useful property to account for phenomena such as blocking [47] or overexpectation [48]. In contrast to the present model, while taking inspiration from RL theory (e.g. using incremental updates), these models are usually far from the classical RL framework. Of significant difference with the present study, most of these models tend to describe the varying intensity of a unique conditioned response and do not account for variations in the actual form of the response, as we do here. In such models, the magazine would not be taken into account and/or taken as part of the context, making it unable to acquire a value for itself nor be the focus of a particular response.

In RL theory, factorization is mainly evoked when trying to overcome the curse of dimensionality [49] (i.e. standard algorithms do not scale well to high dimensional spaces and require too much physical space or computation time). Amongst methods that intend to overcome this problem are value function approximations and Factored Reinforcement Learning. Value function approximations [35], [50], [51] attempt to split problems into orthogonal subproblems making computations easier and providing valuations that can then be aggregated to estimate the value of states. Factored Reinforcement Learning [52]–[54] attempts to find similarities between states so that they can share values, reducing the physical space needed and relies on factored Markov Decision Processes. We also use factored Markov Decision processes, hence the “factored” terminology. However, our use of factored representations serves a different purpose. We do not intend to build a compact value-function nor infer the value of states from values of features but rather make these values compete in the choice for the next action.

Taking advantage of factored representations into classical RL algorithms is at the very heart of the present results. By individually processing stimuli within states (i.e. in the same context, at the same time and same location) and making them compete, the Feature-Model-Free system favours a different policy – oriented towards engaging with the most valued stimuli – (sign-tracking) than would have been favoured by classical algorithms such as Model-Based or Model-Free systems (goal-tracking). Hence, combining a classical RL algorithm with the Feature-Model-Free system enables the model to reproduce the difference in behaviours observed between STs and GTs during an autoshaping procedure. Moreover, by biasing expected optimal behaviours towards cues with motivational values (incentive salience), it is well suited to explain the observed commitment to unnecessary and possibly counter-productive actions (see also [16], [55], [56]). Most of all, it enables the model to replicate the different patterns of dopamine activity recorded with FSCV in the core of the nucleus accumbens of STs and GTs. The independent processing of stimuli leads to patterns of RPE that match those of dopamine activity for STs – a shift of bursts from the US to the CS; and in GTs – a persistence of bursts at both the time of the US and the CS.

### A promising combination

By combining the two concepts of dual learning systems and factored representations in a single model, we are able to reproduce individual variation in behavioural, physiological and pharmacological effects in rats trained using an autoshaping procedure. Interestingly, our approach does not require a deep revision of mechanisms that are extensively used in our current field of research.

While Pavlovian and instrumental conditioning seem entangled in the brain [57], the two major concepts on which rely their respective models, dual learning systems and factored representations, have to our knowledge never been combined into a single model in this field of research.

This approach could contribute to the understanding of interactions between these two classes of learning, such as CRE or Pavlovian-Instrumental Transfer (PIT), where motivation for stimuli acquired via Pavlovian learning modulates the expression of instrumental responses. Interestingly, the Feature-Model-Free system nicely fits with what would be expected from a mechanism contributing to general PIT [58]. It is focused on values over stimuli without regard to their nature [58], it biases and interferes with some more instrumental processes [55], [56], [58] and it is hypothesized to be located in the core of the nucleus accumbens [58]. It would thus be interesting to study whether future simulations of the model could explain and help better formalize these aspects of PIT.

We do not necessarily imply that instrumental and Pavlovian conditioning might rely on a unique model. Rather, we propose that if they were the results of separated systems, they should somehow rely on similar representations and valuation mechanisms, given the strength of the observed interactions.

### Theoretical and practical implications

The proposed model explains the persistent dopamine response to the US in GTs over days of training as a permanent RPE due to the revision of the magazine value during each ITI. Therefore, a prediction of the model is that shortening the ITI should reduce the amplitude of this burst (i.e. there should be less time to revise the value and reduce the size of the RPE); whereas increasing the ITI should increase the amplitude of this burst. Removing the food dispenser during ITI, similar to theoretically suppressing the ITI, should make this same burst disappear. Studying physiological data by grouping them given the duration of the preceding ITI might be sufficient, relatively to noise, to confirm that its duration impacts the amplitude of dopamine bursts. In the current experimental procedure, the ITI is indeed randomly picked in a list of values with an average of 90 sec. Moreover, reducing ITI duration should lead to an increase of the tendency to goal-track in the overall population. Indeed, with a higher value of the food magazine, the Feature-Model-Free system would be less likely to favour sign-tracking over goal-tracking CR. The resulting decrease in sign-tracking in the overall population would be consistent with findings of previous works [59]–[62], where a shorter ITI reduces the observed performance in the acquisition of sign-tracking CRs. Alternatively, it would also be interesting to examine the amplitude of dopamine bursts during the ITI (especially when exploring the food magazine), to determine whether or not physiological responses during this period affect the outcome of the conditioned response.

It would be interesting to split physiological data not only between STs and GTs but also between the stimuli on which the rats started and/or ended focusing on during CS presentation at each trial. This would help to confirm that the pattern of dopamine activity is indeed due to a separate valuation of each stimuli. We would predict that at the time of the US, dopamine bursts during engagement with the lever should be small relatively to dopamine bursts during engagement with the magazine. Moreover, comparing dopamine activity at the time of the CS when engaging with the lever versus the magazine could help elucidate which update mechanism is being used. If activity differs, this would suggest that the model should be revised to use SARSA-like updates, i.e. taking into account the next action in RPE computation. Such a question has already been the focus of some studies on dopamine activity [63]–[65].

There is no available experimental data for the phasic dopaminergic activity of the intermediate group. The model predicts that such a group would have a permanent phasic dopamine burst, i.e. RPE, at US and a progressively appearing burst at CS (see Figure S6). Over training, the amplitude of the phasic dopamine burst at US should decrease until a point of convergence, while at the mean time the response at CS should increase until reaching a level higher than the one observed at US. However, one must note, that the fitting of the intermediate group is not as good as for STs or GTs, as it regroups behaviours that range from sign-tracking to goal-tracking, such that this is a weak prediction.

There is the possibility that regularly presenting the magazine or the lever could, without pairing with food, lead to responses that are indistinguishable from CRs. However, ample evidence suggests that the development of a sign-tracking or goal-tracking CR is not due to this pseudoconditioning phenomenon, but rather a result of learned CS-US associations. That is, experience with lever-CS presentations or with food US does not account for the acquisition of lever-CS induced directed responding [22], [66]. Nonetheless, it should be noted that the current model cannot distinguish between pseudoconditioning CR-like responses and sign-tracking or goal-tracking behaviours. This would require us to introduce more complex MDPs that embed the ITI and can more clearly distinguish between approach and engagement.

### Limitations

The Feature-Model-Free system presented in this article was designed as a proof of concept for the use of factored representations in computational neuroscience. In its present form it updates the value of one feature (the focused one) at a time, and this is sufficient to account for much of the experimental data. It does not address whether multiple features could be processed in parallel, such that multiple synchronized, but independently computed, signals would update distinct values relative to the attention paid to the associated features. Further experiments should be performed to confirm this hypothesis. Subsequently, using factored representations in the Model-Based system was not necessary to account for the experimental data and the question remains whether explaining some phenomena would require it.

While using factored representations, our approach still relies on the discrete-time state paradigm of classical RL, where updates are made at regular intervals. Although such simplification can explain the set of data considered here, one would need to extend this to continuous time if one would like to also model experimental data where rats take more or less time to initiate actions that can vary in duration [14]. The present model, which does not take timing into consideration, cannot account for the fact that STs and GTs both come to approach their preferred stimuli faster and faster as a function of training nor does it make use of the variations of ITI duration. Our attempt to overcome this limitation using the MDP framework was unsuccessful. Focusing on features, it becomes more tempting to deal with the timing of their presence, a property that is known to be learned and to have some impact on behaviours [61], [67]–[69].

Moreover, in the current model, we did not attempt to account for the conditioned orienting responses (i.e. orientation towards the CS) that both STs and GTs exhibit upon CS presentation [25]. However, we hypothesize that such learned orienting responses could be due to state discrimination mechanisms that are not included in the model, and would be better explained with partial observability and actions dedicated to collect information. This is beyond the scope of the current article, but is of interest for future studies.

As evident by the only partial reproduction of the flupentixol effects on the expression of sign- and goal-tracking behaviours, the model is limited by the use of the softmax action-selection mechanism, which is widely used in computational neuroscience [16]–[19], [32], [34]–[36]. In the model, all actions are equal – there is no action with a specific treatment – and the action-selection mechanism necessarily selects an action at each time step. Any reduction in the value of one action favours the selection of all other actions in proportion to their current associated values. In reality, however, blocking the expression of an action would certainly lead mainly to inactivity rather than necessarily picking the alternative and almost never expressed action. One way of improving the model in this direction could be to replace the classical softmax function by a more realistic model of action selection in the basal ganglia (e.g. [70]). In such a model, no action is performed when no output activity gets above a certain threshold. Humphries et al. [32] have shown that changing the exploration level in a softmax function can be equivalent to changing the level of tonic dopamine in the basal ganglia model of Gurney et al. [70]. Interestingly, in the latter model, reducing the level of tonic dopamine results in difficulty in initiating actions and thus produces lower motor behaviour, as is seen in Parkinsonian patients and as can be seen in rats treated with higher doses of flupentixol [14]. Thus a natural sequel to the current model would be to combine it with a more realistic basal ganglia model for action selection.

We simulated the effect of flupentixol as a reduction of the RPE in the learning processes of Model-Free systems to parallel its blockade of the dopamine receptors. While this is sufficient to account for the pharmacological results previously reported [14], it fails to account for some specific aspects that have more recently emerged. Mainly, it is unable to reproduce the instant decreased engagement observed at the very first trial after post-training local injections of flupentixol [25]. Our current approach requires re-learning to see any impact of flupentixol. A better understanding of the mechanisms that enable instant shifts in motivational values, by shifts in the motivational state [71] or the use of drugs [14], [25], might be useful to extend the model on such aspects.

We also tried to model the effect of flupentixol on RPEs with a multiplicative effect, as it would have accounted for an instant impact on behaviour. However, it failed to account for the effects of flupentixol on learning of the sign-tracking CRs, as a multiplicative effect only slowed down learning but did not disrupt it. How to model the impact of flupentixol, and dopamine antagonists or drugs such as cocaine remains an open question (e.g. see [72], [73]).

Finally, our work does not currently address the anatomical counterpart of 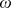 at the heart of the model, nor the regions of the brain that would match the current Model-Based system and the Feature-Model-Free system. Numerous studies have already discussed the potential substrates of Model-Based/Model-Free systems in the prefrontal cortex/dorsolateral striatum [74], or the dorsomedial and dorsolateral striatum [33], [75]–[78]. The weighted sum integration may suggest a crossed projection of brains regions favouring sign- and goal-tracking behaviours (Model-Based and Feature-Model-Free systems) into a third one. We postulate there is a difference in strength of “connectivity” between such regions in STs vs GTs [79]. Further, one might hypothesize that the core of the nucleus accumbens contributes to the Feature-Model-Free system. The integration and action selection mechanisms would naturally fit within the basal ganglia, stated to contribute to such functions [32], [80]–[82].

### Conclusion

Here we have presented a model that accounts for variations in the form of Pavlovian conditioned approach behaviour seen during autoshaping in rats; that is, the development of a sign-tracking vs goal-tracking CR. This works adds to an emerging set of studies suggesting the presence and collaboration of multiple RL systems in the brain. It questions the classical paradigm of state representation and suggests that further investigation of factored representations in RL models of Pavlovian and instrumental conditioning experiments may be useful.

## Methods

### Modelling the autoshaping experiment

In the classical reinforcement learning theory [1], tasks are usually described as Markov Decision Processes (MDPs). As the proposed model is based on RL algorithms, we use the MDP formalism to computationally describe the Pavlovian autoshaping procedure used in all simulations.

An MDP describes the interactions of an agent with its environment and the rewards it might receive. An agent being in a state 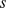 can execute an action 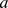 which results in a new state 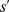 and the possible retrieval of some reward 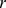. More precisely, an agent can be in a finite set of states 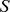, in which it can perform a finite set of discrete actions 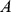, the consequences of which are defined by a transition function 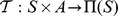, where 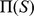 is the probability distribution 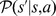 of reaching state 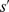 doing action 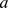 in state 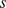. Additionally, the reward function 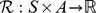 is the reward 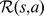 for doing action 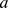 in state 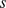. Importantly, MDPs should theoretically comply with the Markov property: the probability of reaching state 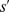 should only depend on the last state 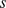 and the last action 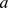. An MDP is defined as episodic if it includes at least one state which terminates the current episode.

Figure 1 shows the deterministic MDP used to simulate the autoshaping procedure. Given the variable time schedule (30–150s) and the net difference observed in behaviours in inter-trial intervals, we can reasonably assume that each experimental trial can be simulated with a finite horizon episode.

The agent starts from an empty state (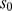) where there is nothing to do but explore. At some point the lever appears (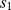) and the agent must make a critical choice: It can either go to the lever (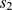) and engage with it (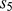), go to the magazine (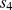) and engage with it (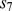) or just keep exploring (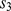,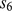). At some point, the lever is retracted and food is delivered. If the agent is far from the magazine (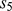,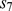), it first needs to get closer. Once close (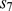), it consumes the food. It ends in an empty state (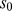) which symbolizes the start of the inter-trial interval (ITI): no food, no lever and *an empty but still present magazine*.

The MDP in Figure 1 is common to all of the simulations and independent of the reinforcement learning systems we use. STs should favour the red path, while GTs should favour the *shorter* blue path. All of the results rely mainly on the action taken at the lever appearance (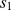), when choosing to go to either the lever, the magazine, or to explore. Exploring can be understood as not going to the lever nor to the magazine.

To fit with the requirements of the MDP framework, we introduce two limitations in our description, which also simplify our analyses. We assume that engagement is necessarily exclusive to one or no stimulus, and we make no use of the precise timing of the procedure – the ITI duration nor the CS duration – in our simulations.

#### Inter-trial interval (ITI)

While the MDP does not model the ITI, the results regarding physiological data rely partially on its presence. Extending the MDP with a set of states to represent this interval would increase the complexity of the MDP and the time required for simulations. The behaviour that could have resulted from such an extension is easily replaced by applying the following formula at the beginning of each episode:
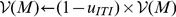
(1)where the parameter 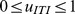 reflects the interaction with the magazine that occurred during the ITI. A low 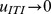 symbolizes a low interaction and therefore a low revision of the value associated to the magazine. A high 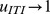 symbolizes a strong exploration of the magazine during the inter-trial interval and therefore a strong decrease in the associated value due to unrewarded exploration.

### Model

The model relies on the architecture shown in Figure 2. The main idea is to combine the computations of two distinct reinforcement learning systems to define what behavioural response is chosen at each step.

#### Model-Based system (MB)

The first system is Model-Based [1], and classically relies on a transition function 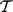 and a reward function 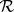 which are learned by experience given the following rules:
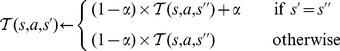
(2)

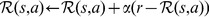
(3)where the learning rate 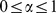 classically represents the speed at which new experiences replace old ones. Using a learning rate rather than counting occurrences is a requirement for accordance with the incremental expression of the observed behaviours. This can account for some resistance or uncertainty in learning from new experiences.

Given this model, an action-value function 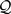 can then be computed with the following classical formula:
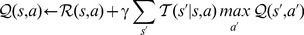
(4)where the discount rate 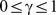 classically represents the preference for immediate versus distant rewards. The resulting Advantage function 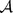 [83], [84], the output of the first system, is computed as follows:
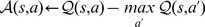
(5)It defines the (negative) advantage of taking action 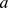 in state 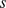 relatively to the optimal action known. The optimal action therefore has an advantage value of 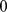.

In terms of computation, the advantage function could be replaced by the action-value function without changing the simulation results (we only compare 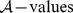 over the same state and therefore 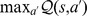 is constant whatever the action). It has been used in preceding works dealing with interactions between instrumental and Pavlovian conditioning [16], [84] and we kept it for a better and more straightforward comparison with variants of the model that were directly inspired by these preceding works.

#### Feature-Model-Free system (FMF)

**Figure 10:**
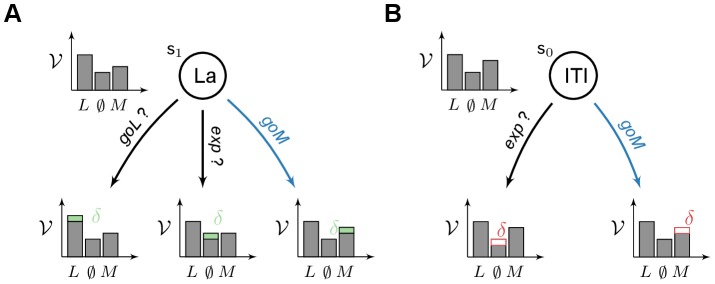
Characteristics of the Feature-Model-Free system. (**A**) Focusing on a particular feature. The Feature-Model-Free system relies on a value function 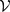 based on features. Choosing an action (e.g. *goL*, *goM* or *exp*), defines the feature it is focusing on (e.g. *L*ever, *M*agazine or nothing 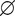). Once the action is chosen (e.g. *goM* in blue), only the value of the focused feature (e.g. 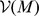) is updated by a standard reward prediction error, while leaving the values of the other features unchanged. (**B**) Feature-values permit generalization. At a different place and time in the episode, the agent can choose an action (e.g. *goM* in blue) focusing on a feature (e.g. *M*) that might have already been focused on. This leads to the revision of the same value (e.g. 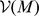) for two different states (e.g. 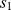 and 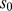). Values of features are shared amongst multiple states.

A state is generally described by multiple features. Animals, especially engaged in a repetitive task, might not pay attention to all of them at once. For example, when the lever appears and a rat decides to engage with the magazine, it focuses primarily on the magazine while ignoring the lever, such that it could update a value associated to the magazine but leave intact any value related to the lever (see Figure 10 A). Although this could be related to an attentional process that bias learning, we do not pretend to model attention with such a mechanism.

Relying on this idea, the second system is a revision of classical Model-Free systems which is based on features rather than states. It relies on a value function 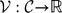 based on a set of features 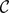, which is updated with an RPE:
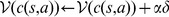
(6)

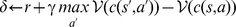
where 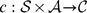 is a feature-function that returns the feature 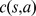 the action 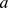 was focusing on in state 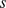 (see Table S2; Figure 1 also embeds the features returned by 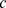 for each action and state). One could argue that this feature-function, defined *a priori*, introduces an additional requirement relative to classical Model-Free systems. This is a weak requirement since this function is straightforward when actions, instead of being abstractly defined, are described as interactions towards objects in the environment. This function simply states that, for example, when pressing a lever, the animal is focusing on the lever rather than on the magazine. Similar to 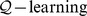, we assume that the future action to be chosen is the most rewarding one. Therefore, the value chosen for the reached state 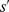, in the computation of the RPE, is the highest value reachable by any possible future action 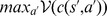.

Classical Model-Free systems do not permit generalization in their standard form: even when two states share most of their features, updating the value of one state leaves the value of the other untouched. This new system overcomes such limitation (see Figure 10 B). In Feature-Model-Free Reinforcement Learning, multiple states in time and space can share features and their associated values. For example, while in ITI, rats tend from time to time to explore the magazine [22], [26], which might lead them to revise any associated value, which can also be used when the lever appears. Therefore, actions in ITIs might impact the rest of the experiment.

In the simulated experiment (see Figure 1), this generalization phenomenon happens as follows: Assuming that the simulated rat was engaging the magazine (eng) before food delivery (from 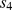 to 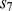), then the value 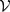 of 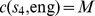 is updated with the following 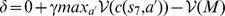. As the best subsequent action (and, for simplification, the only possible one) is to consume the food (in 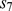), it results in a positive 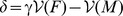. During ITI (which in the MDP is simulated by the 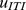 parameter), if the simulated rat checks the magazine (goM) and finds no food, then 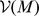 is revised with a negative 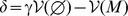 (Figure 10 B). The value 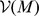 is therefore revised at multiple times in the experiment and, for example, a decrease of value during ITI has an impact on the choice of engaging with the magazine (goM) at lever appearance.

Processing features rather than states and the generalization that results from it is a key mechanism of the presented model. It makes the system favour a different path than the one favoured by classical reinforcement learning systems.

Contrary to what the system suggests, it is almost certain that rats might handle multiple features at once and could simultaneously update multiple values. We present here a version without such capacity since it is not required in the simulated experiments and simplifies its understanding.

#### Integration

The Feature-Model-Free system accounts for motivational bonuses 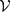 that impact values 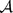 computed by the Model-Based system. The integration of these values is made through a weighted sum:
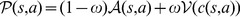
(7)where 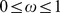 is a combination parameter which defines the importance of each system in the overall model. 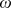 is equivalent to the responsibility signal in Mixture of Experts [35], [85]. We want to emphasize that the two systems are not in simple competition, and it is not the case that there is a unique system acting at a time. Rather, they are both active and take part in the decision proportionally to the fixed parameter 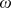. A simple switch between systems would not account for the full spectrum of observed behaviours ranging from STs to GTs [26].

#### Action selection

We use a softmax rule on the integrated values 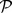 to compute the probability to select an action 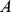 in state 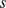:
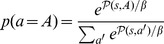
(8)where 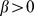 is the selection temperature that defines how probabilities are distributed. A high temperature (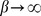) makes all actions equiprobable, a low one makes the most rewarding action almost exclusive.

#### Impact of flupentixol

When simulating the pharmacological experiments, namely the impact of flupentixol, a parameter 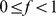 is used to represent the impact of flupentixol on parts of the model.

As a dopamine receptor antagonist, we model the impact of flupentixol on phasic dopamine by revising any RPE 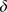 used in the model given the following formula:
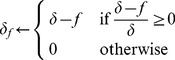
(9)where 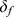 is the new RPE after flupentixol injection. The impact is filtered (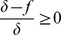) such that flupentixol injection could not lead to negative learning when the RPE was positive, but at most block it (i.e. the sign of 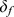 cannot be different from the one of 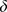). With a low 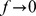, the RPE is not affected (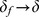). A high 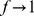 reduces the RPE, imitating a blockade of dopamine receptors.

Various studies (e.g. [32]) also suggest that tonic dopamine has an impact on action selection such that any decrease in dopamine level results in favouring exploration over exploitation. We therefore simulated the effect of flupentixol on action selection by revising the selection temperature given the following formula:
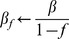
(10)where 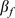 is the new selection temperature, and 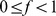 represents the strength of the flupentixol impact. A strong 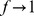, which represents an effective dose of flupentixol, favours a high temperature 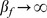 and therefore exploration. A low 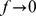, i.e. a low dose or an absence of flupentixol, leaves the temperature unaffected: 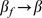.

For the first pharmacological experiment (Effects of systemic flupentixol administration on the learning of sign- and goal-tracking behaviours) both the impact on the softmax and on the RPE were activated, as the flupentixol was injected systemically and assumed to diffuse in the whole brain. For the second experiment (Effects of local flupentixol administration on the expression of sign- and goal-tracking behaviours) only the impact on the RPE was activated, as the flupentixol was injected locally in the core of the nucleus accumbens. We hypothesize that the Feature-Model-Free system relies in the core of the nucleus accumbens whereas the selection process (softmax) does not.

#### Initialization

In the original experiments [14], [20], prior to the autoshaping procedure, rats are familiarized with the Skinner box and the delivery of food into the magazine. While the MDP does not account for such pretraining, we can initialize the model with values (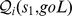, 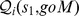 and 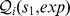) that reflect it (see the estimation of the model parameters). These initial values can be seen as extra parameters common to the model and its variants.

### Variants

**Figure 11:**
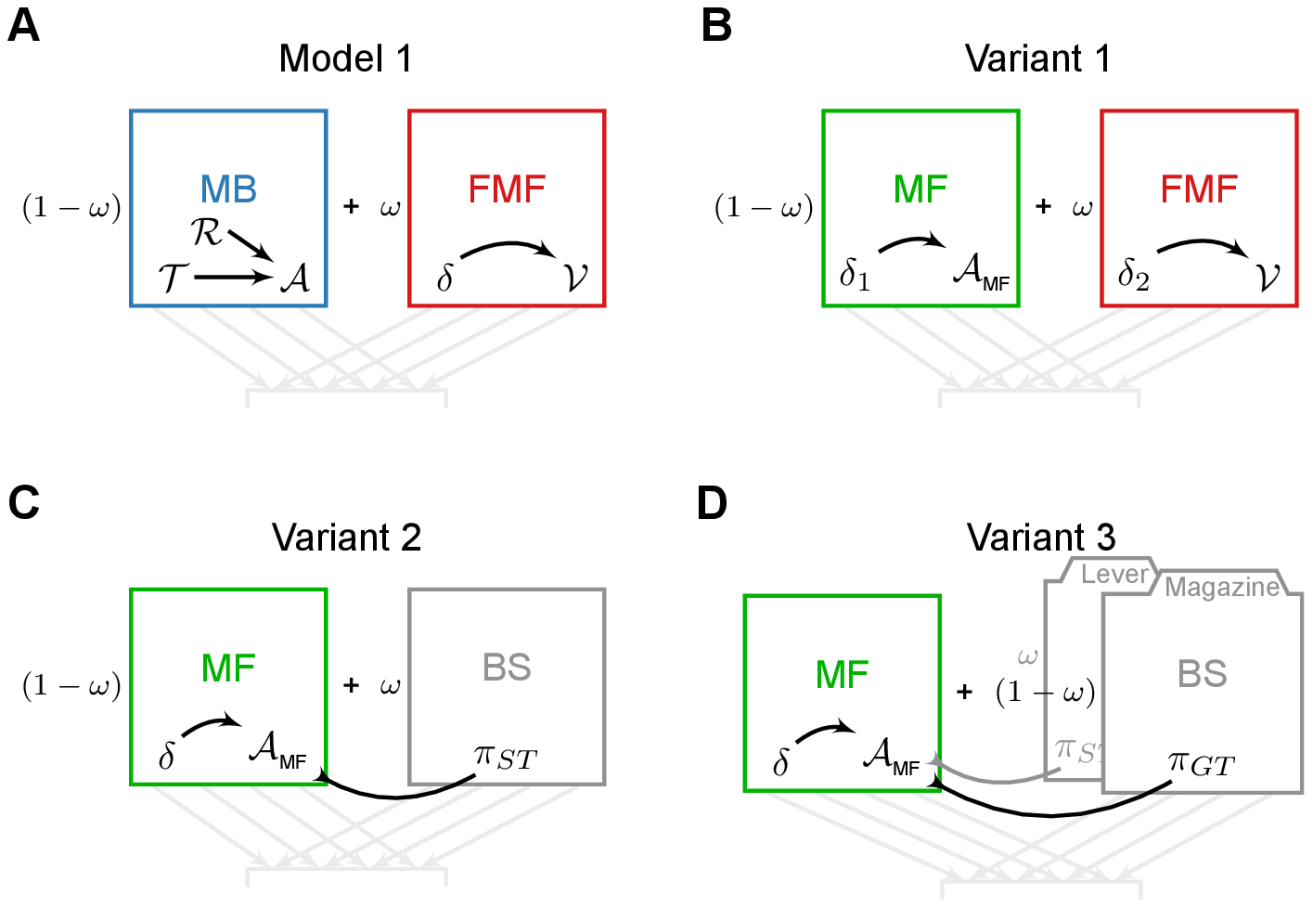
Systems combined in the model and the variants. Variants of the model rely on the same architecture (described in Figure 2) and only differ in the combined systems. Colours are shared for similar systems. (**A**) The model combines a Model-Based system (MB, in blue) and a Feature-Model-Free (FMF, in red) system. (**B**) Variant 1 combines a Model-Free system (MF, in green) and a Feature-Model-Free system. (**C**) Variant 2 combines a Model-Free system and a Bias system (BS, in grey), that relies on values from the Model-Free system. (**D**) Variant 3 combines a Model-Free system and two Bias systems, that rely on values from the Model-Free system. Variant 4 is not included as it failed to even reproduce the autoshaping behavioural results.

Given the modular architecture of the model, we were able to test different combinations of RL systems. Their analysis underlined the key mechanisms required for reproducing each result (see Figures S1, S2, S4 and S5). Figure 11 (B, C and D) schematically represents the analysed variants.

Most of the results rely on the action taken by the agent at the lever appearance. The action taken results from the values 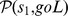, 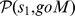 and 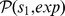, the computation of which differs in each of the variants described below.

#### Variant 1 : Model-Free/Feature-Model-Free

Variant 1 was tested to assert the necessity of the Model-Based system as part of the model to reproduce the results. Thus in Variant 1, the Model-Based system is replaced by a classical Model-Free system, Advantage learning [83], [84], while the Feature-Model-Free system remains unchanged (see Figure 11 B).

In such a Model-Free system, the action-value function 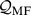 is updated online according to the transition just experienced. At each time step the function is updated given an RPE 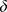 that computes the difference between the observed and the expected value, as follows:
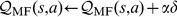
(11)

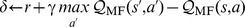



Computation of the associated Advantage function 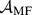 follows Equation (5). This model computes integrated values as follows:
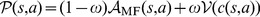
(12)


It is important to note that while Equation (12) looks similar to Equation (7), the Advantage function is computed by a Model-Based system in the model (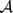) and a Model-Free system in this variant (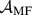), leading to very different results on pharmacological experiments.

#### Variant 2 : Asymmetrical

Inspired by a work from Dayan et al. [16], Variant 2 combines a classical Advantage learning system [83], [84] with some Bias system taking its values directly from the other system (see Figure 11 C). This system computes the integrated values as follows:
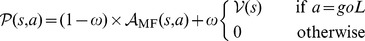
(13)


It asymmetrically gives a bonus to the path that should be taken by STs. In slight discrepancy with the original model, it uses the maximum value over action-value function 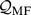 as the value function 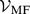 used to compute the advantage function. Hence, there is a single RPE computed at each step.

#### Variant 3 : Symmetrical

In the same line as Variant 2, Variant 3 symmetrically gives a bonus to both paths using a classical Advantage learning system in combination with a Pavlovian system. This system computes the integrated values as follows:
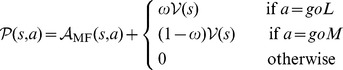
(14)


This model does not exactly fit Equation (7) of the general architecture. It is based on 3 systems, where the real competition is between the two bias systems, whereas the Model-Free system is mainly used to compute the values used by the two others (see Figure 11 D). The rest of the architecture is not impacted.

#### Variant 4 : Model-Based/Model-Free

Variant 4 was developed to confirm the necessity of a feature-based system. It combines two advantage functions computed from a Model-Based (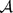) and a Model-Free (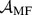) system.
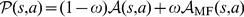
(15)


While computed differently, both advantage functions will eventually converge to the same optimal values [1] making both systems favouring the same optimal policy. Note that 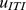 cannot be used in this variant as there exists no value over the magazine itself. While varying the parameters might slow down learning or make the process more exploratory, this could never lead to sign-tracking as both systems, whatever the weighting, would favour goal-tracking. As such, Variant 4 is unable to even account for the main behavioural results in the autoshaping procedure (see Figure S8).

Given that all the subsequent simulated results relies on a correct reproduction of the default behaviours, this variant was not investigated further and is not compared to the other variants in supplementary results figures.

### Estimating the model parameters

The model relies on model-specific parameters (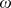, 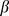, 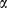 and 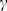) and experience-specific parameters (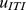, 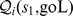, 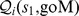 and 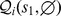). If the model were used to simulate a different experiment, the model-specific parameters would be the same while different experience-specific parameters might be required. For an easier analysis and a simpler comparison between the model and its variants, we reduce the number of parameters by sharing parameters with identical meanings amongst systems (i.e. both systems within the model share values for their learning rates 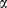 and discount rates 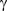, rather than having independent parameter values).

Due to the number of parameters, finding the best values to qualitatively fit the experimental data cannot be done by hand. Using a genetic algorithm makes it possible to optimize the search of suitable values for the parameters.

Parameter values were retrieved by fitting the simulation of the probabilities to engage either the lever or the magazine with the experimental data of one of the previous studies [21]. No direct fitting was intended on other experimental data. Hence, a single set of values was used to simulate behavioural, physiological and pharmacological data.

If for a variant, the optimization algorithm fails to fit the experimental data, it suggests that whatever the values, the mechanisms involved cannot explain the behavioural data (Variant 4).

Probabilities to engage the lever or the magazine were taken as independent objectives of the algorithm, since fitting sign-tracking probabilities is easier than fitting goal-tracking probabilities. For each objective, the fitness function is computed as the least square errors between the experimental and simulated data. Parameter optimization is done with the multi-objective genetic algorithm NSGA-II [86]. We used the implementation provided by the Sferes 2 framework [87]. All parameters required for reproducing the behavioural data were fitted at once.

For NSGA-II, we arbitrarily use a population of 200 individuals and run it over 1000 generations. We use a polynomial mutation with a rate of 0.1, and simulate binary cross-overs with a rate of 0.5. We select the representative individual, to be displayed in figures, from the resulting Pareto front by hand, such that it best visually fits the observed data.

To confirm that 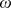 is the key parameter of the model, we additionally tried to fit the whole population at once (i.e. sharing all parameter values in agents but 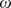) and we were still able to reproduce the observed tendencies of sign- and goal-tracking in the population (see Figure S7 A,B) and the resulting different phasic dopaminergic patterns (see Figure S7 C,D).

It is however almost certain that each subgroup does not express the exact same values for the other parameters. Removing such constraint by fitting each subgroup separately, indeed provides better results. Results presented in this article are based on such separate fitting.

## Supporting Information

Figure S1 **Comparison of variants of the model on simulations of autoshaping experiment.** Legend is as in Figure 5 (C,D). Simulation parameters for STs (red), GTs (blue) and IGs (white) in the model (**A**), Variant 1 (**B**), Variant 2 (**C**) and Variant 3 (**D**) are summarized in Table S1. All variants reproduce the spectrum of behaviours ranging from sign-tracking to goal-tracking.
(TIFF)

Figure S2 **Comparison of variants of the model on incentive salience and Conditioned Reinforcement Effect intuitions.** Legend is as in Figure 6. Simulation parameters for STs (red), GTs (blue) and IGs (white) are summarized in Table S1. Variant 2 (**C**) relying on asymmetrical bonuses given only to sign-tracking cannot reproduce the attribution of a motivational value by the second system to both the lever and the magazine. Others (**A,B,D**) attribute values to both stimuli and parallels the supposed acquisition of motivational values by stimuli, i.e. incentive salience. All variants are able to account for a Conditioned Reinforcement Effect more pronounced in STs than in GTs.
(TIFF)

Figure S3 **Comparison of variants of the model on simulations of patterns of dopaminergic activity.** Legend is as in Figure 7 (C,D). Simulation parameters for STs (left) and GTs (right) are summarized in Table S1. The model (**A**) and Variant 1 (**B**) can reproduce the difference observed in dopaminergic patterns of activity in STs versus GTs. Other variants (**C,D**) fail to do so, given that the classical Model-Free system propagates the RPE from food delivery to lever appearance on all pathways of the MDP.
(TIFF)

Figure S4 **Comparison of variants on simulations of the effect of systemic injections of flupentixol.** Legend is as in Figure 8 (C,D). Simulation parameters for STs (left) and GTs (right) are summarized in Table S1. Only the Model (**A**) can reproduce the difference in response to injections of flupentixol observed in STs versus GTs. All variants (**B,C,D**) fail to do so, given that they only rely on Model-Free, i.e. RPE-dependent, mechanisms that are blocked by flupentixol.
(TIFF)

Figure S5 **Comparison of variants on simulations of the effect of post injections of flupentixol.** Legend is as in Figure 9 (C,D). Simulation parameters for groups of rats composing the population are summarized in Table S1. Variants 2 (**C**) and 3 (**D**), accounting for sign- and goal-tracking using a single set of values, have a similar impact of flupentixol on both behaviours, leaving relative probabilities to engage with lever and magazine unaffected. Variant 1 (**B**) uses different systems, thus flupentixol impacts sign-tracking in the model in the same way as it does in experimental data. However, given that both systems rely on RPE-dependent mechanisms, the impact is not as visible as in the model (**A**).
(TIFF)

Figure S6
**Prediction of the model about expected patterns of dopaminergic activity in intermediate groups.** Data are expressed as mean 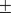 S.E.M. Average RPE computed by the Feature-Model-Free system in response to CS and US presentation for each session of conditioning in the intermediate group. Simulated group is defined as in Figure 5.(TIFF)

Figure S7
**Behavioural and physiological simulations of autoshaping with shared parameter values across STs, GTs and IGs.** (**A,B**) Legend is as in Figure 5 (C,D). Reproduction of the respective tendencies to sign- and goal-track of STs (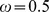), IGs (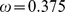) and GTs (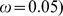) using a single set of parameters (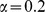, 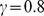, 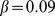, 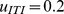, 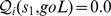, 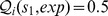 and 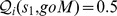). (**C,D**) Legend is as in Figure 7 (C,D). Reproduction of the different patterns of phasic dopaminergic activity in STs and GTs using the same single set of parameters. By simply varying the 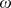 parameter, the model can still qualitatively reproduce the observations in experimental data.(TIFF)

Figure S8 **Simulation of autoshaping experiment for Variant 4.** Legend is as in Figure 5 (C,D). Simulation for parameters STs (red), GTs (blue) and IGs (white) in the Variant 4 are summarized in Table S1. Variant 4 is not even able to reproduce the main behavioural data.
(TIFF)

Table S1
**Summary of parameters used in simulations.** Parameters retrieved by optimisation with NSGA-II and used to produce the results presented in this article for the model and its variants. Parameters for STs, GTs and IGs were optimized separately (A,B,C,D,E). To confirm that 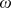 is the key parameter of the model, we also optimized parameters for STs, GTs and IGs by sharing all but the 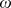 parameter (F) to produce Figure S7.(TIFF)

Table S2
**Definition of feature-function** 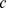 **.** Stimuli (*L*ever, *M*agazine, *F*ood or 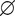) returned by the feature-function 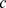 for each possible state-action pair 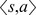 in the MDP described in Figure 1. The feature-function simply defines the stimulus that is the focus of an action in a particular state.(TIFF)
