# Supplementary figures and images for: Modelling Individual Differences in the Form of Pavlovian Conditioned Approach Responses: A Dual Learning Systems Approach with Factored Representations
Source: PLoS Comput Biol. 2014 Feb 13;10(2):e1003466. doi: 10.1371/journal.pcbi.1003466 (PMC3923662; doi:10.1371/journal.pcbi.1003466)

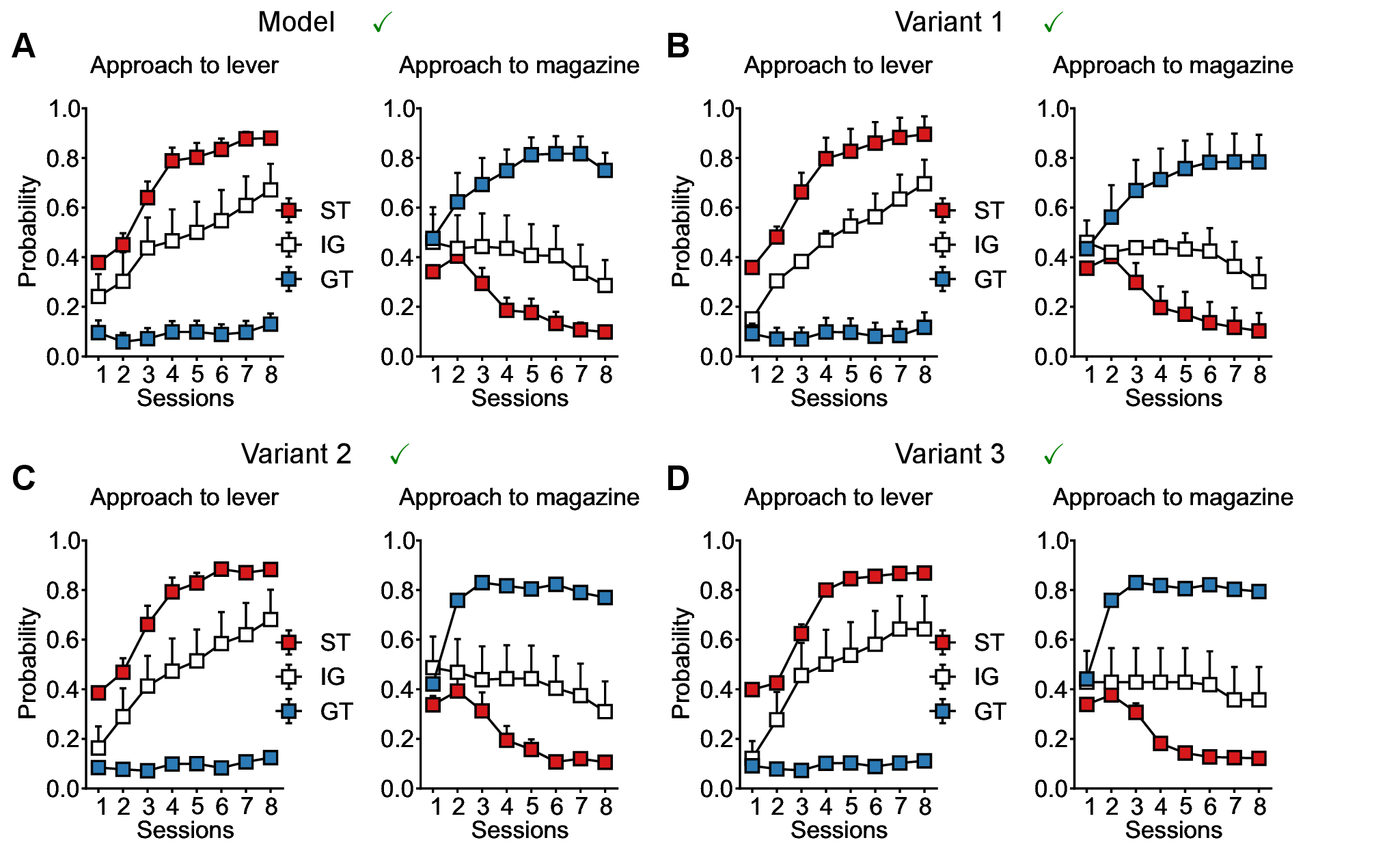

Supplement: Figure S1 — Comparison of variants of the model on simulations of autoshaping experiment. Legend is as in Figure 5 (C,D). Simulation parameters for STs (red), GTs (blue) and IGs (white) in the model (A), Variant 1 (B), Variant 2 (C) and Variant 3 (D) are summarized in Table S1. All variants reproduce the spectrum of behaviours ranging from sign-tracking to goal-tracking. (TIFF) [file pcbi.1003466.s001.tiff]

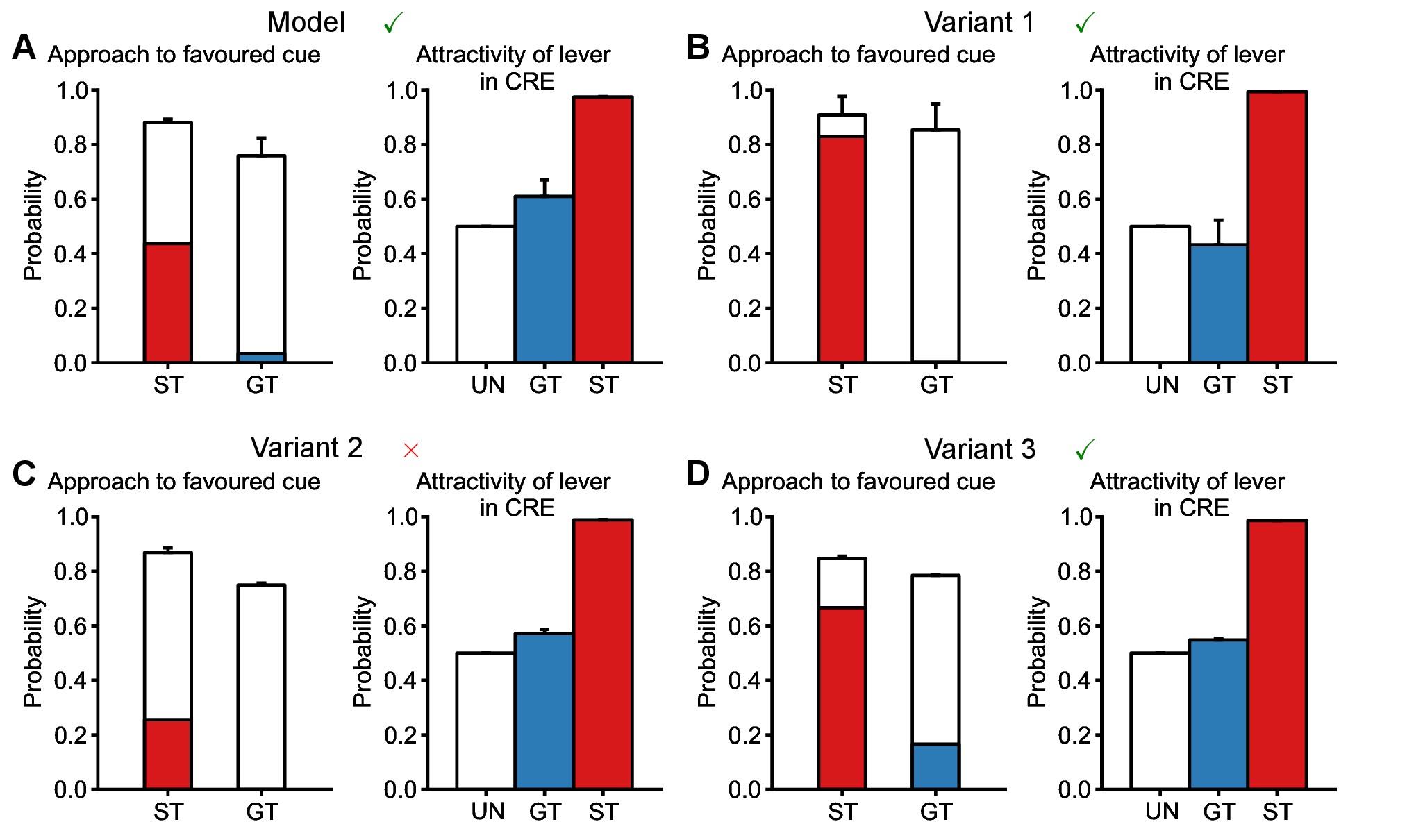

Supplement: Figure S2 — Comparison of variants of the model on incentive salience and Conditioned Reinforcement Effect intuitions. Legend is as in Figure 6. Simulation parameters for STs (red), GTs (blue) and IGs (white) are summarized in Table S1. Variant 2 (C) relying on asymmetrical bonuses given only to sign-tracking cannot reproduce the attribution of a motivational value by the second system to both the lever and the magazine. Others (A,B,D) attribute values to both stimuli and parallels the supposed acquisition of motivational values by stimuli, i.e. incentive salience. All variants are able to account for a Conditioned Reinforcement Effect more pronounced in STs than in GTs. (TIFF) [file pcbi.1003466.s002.tiff]

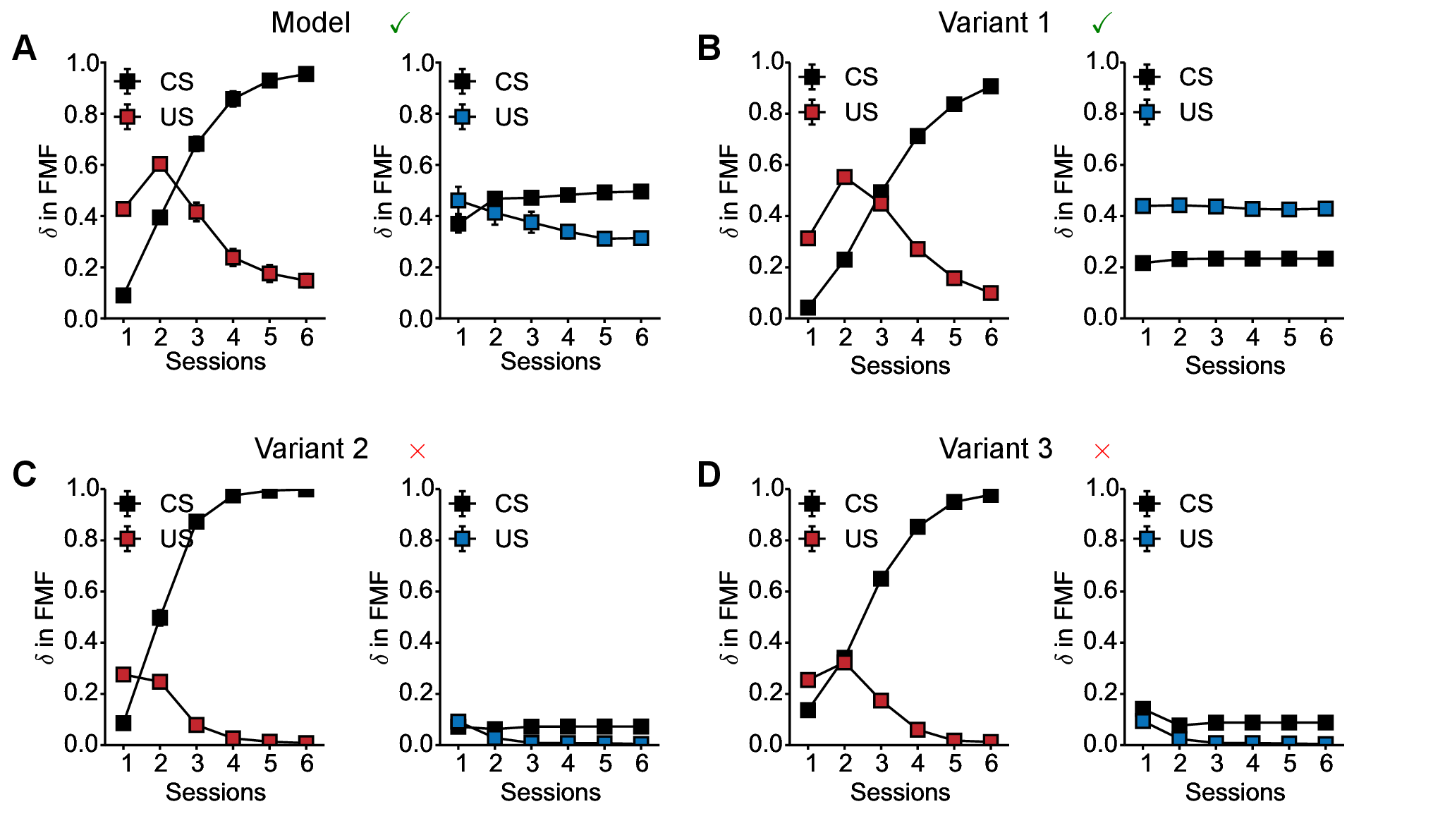

Supplement: Figure S3 — Comparison of variants of the model on simulations of patterns of dopaminergic activity. Legend is as in Figure 7 (C,D). Simulation parameters for STs (left) and GTs (right) are summarized in Table S1. The model (A) and Variant 1 (B) can reproduce the difference observed in dopaminergic patterns of activity in STs versus GTs. Other variants (C,D) fail to do so, given that the classical Model-Free system propagates the RPE from food delivery to lever appearance on all pathways of the MDP. (TIFF) [file pcbi.1003466.s003.tiff]

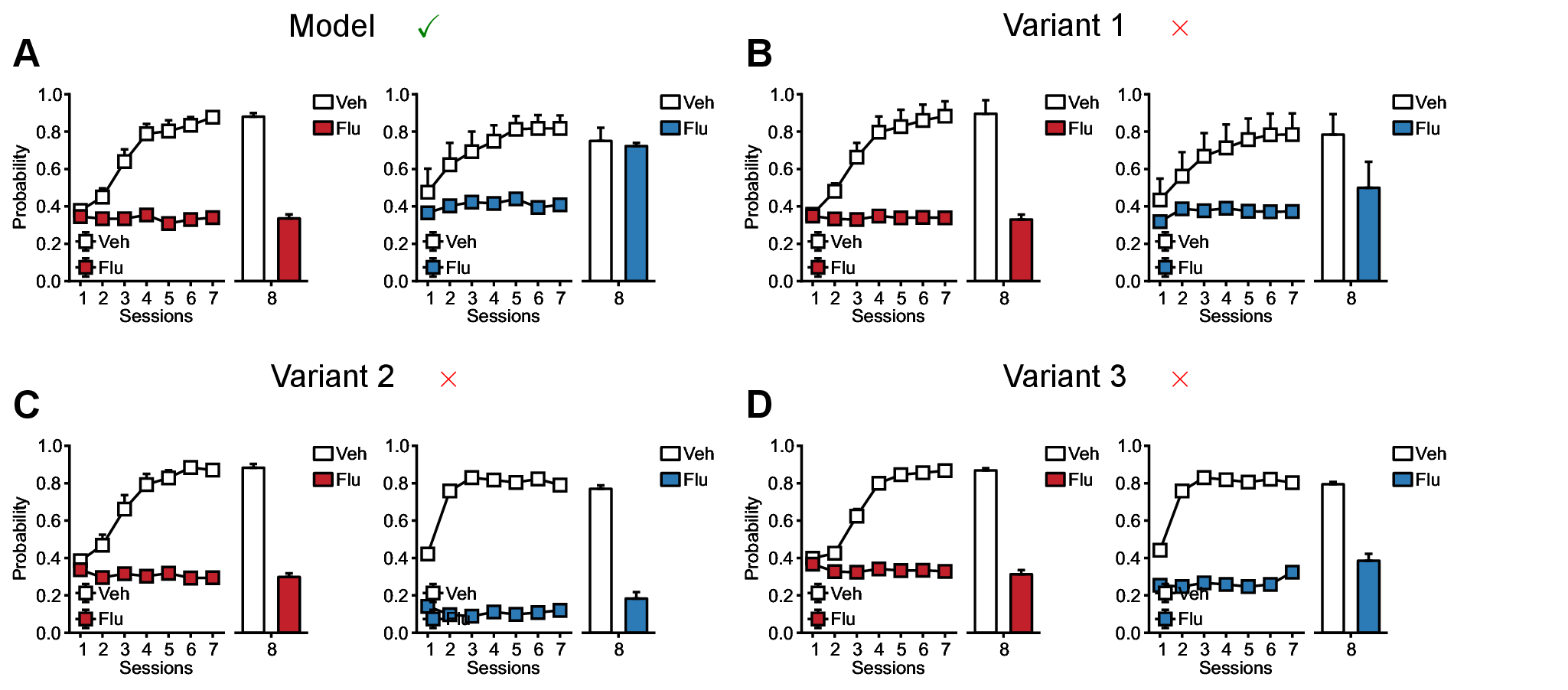

Supplement: Figure S4 — Comparison of variants on simulations of the effect of systemic injections of flupentixol. Legend is as in Figure 8 (C,D). Simulation parameters for STs (left) and GTs (right) are summarized in Table S1. Only the Model (A) can reproduce the difference in response to injections of flupentixol observed in STs versus GTs. All variants (B,C,D) fail to do so, given that they only rely on Model-Free, i.e. RPE-dependent, mechanisms that are blocked by flupentixol. (TIFF) [file pcbi.1003466.s004.tiff]

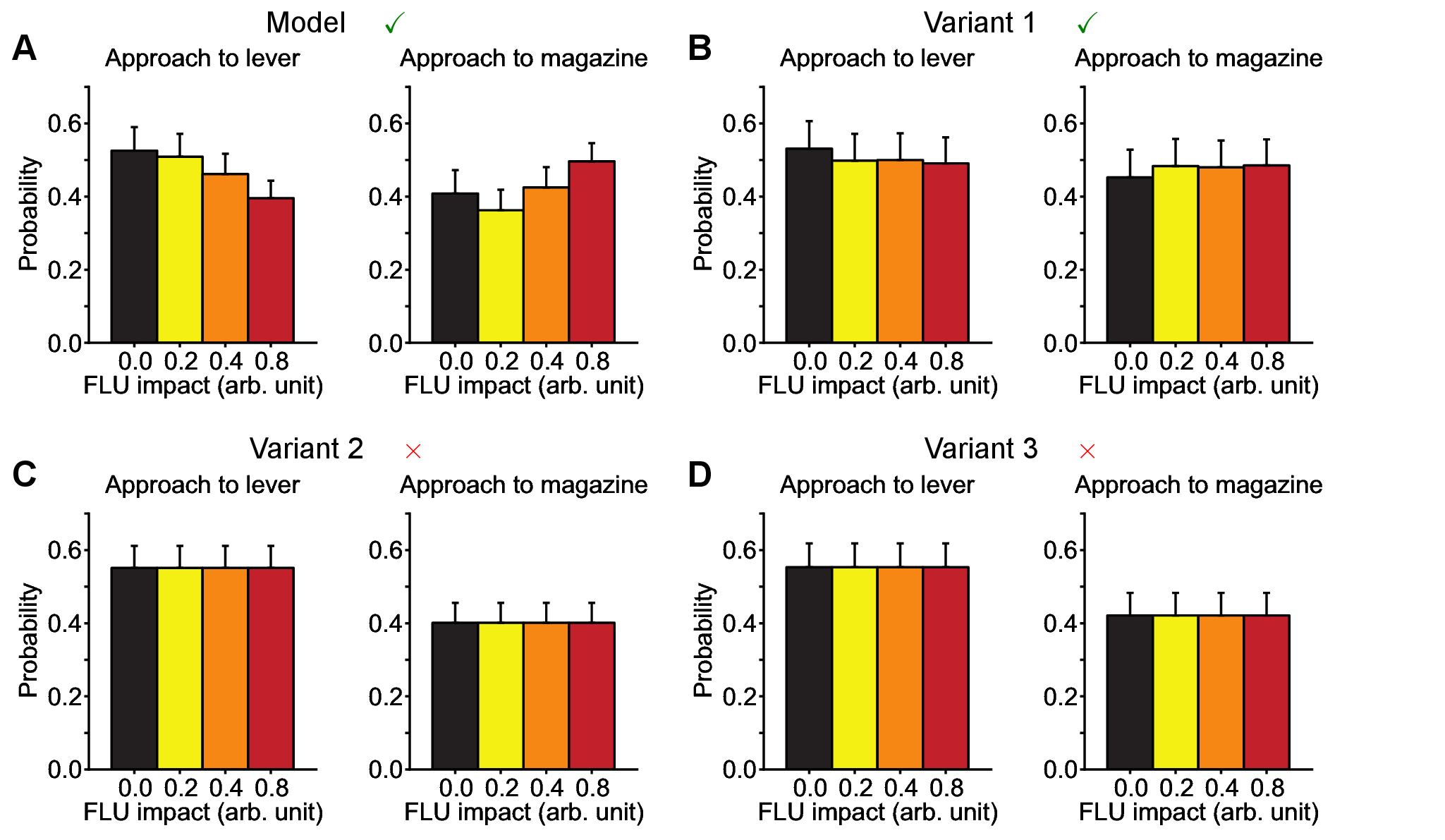

Supplement: Figure S5 — Comparison of variants on simulations of the effect of post injections of flupentixol. Legend is as in Figure 9 (C,D). Simulation parameters for groups of rats composing the population are summarized in Table S1. Variants 2 (C) and 3 (D), accounting for sign- and goal-tracking using a single set of values, have a similar impact of flupentixol on both behaviours, leaving relative probabilities to engage with lever and magazine unaffected. Variant 1 (B) uses different systems, thus flupentixol impacts sign-tracking in the model in the same way as it does in experimental data. However, given that both systems rely on RPE-dependent mechanisms, the impact is not as visible as in the model (A). (TIFF) [file pcbi.1003466.s005.tiff]

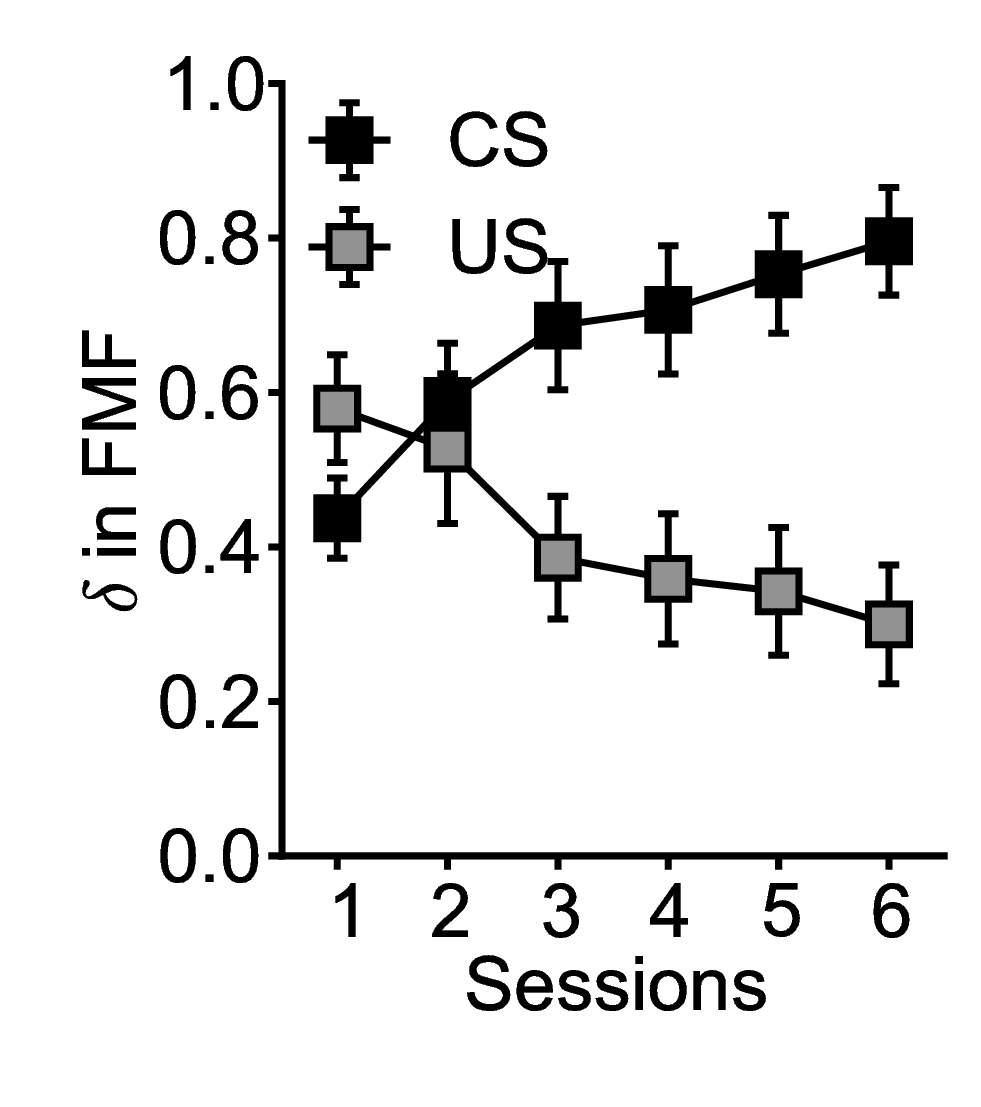

Supplement: Figure S6 — Prediction of the model about expected patterns of dopaminergic activity in intermediate groups. Data are expressed as mean S.E.M. Average RPE computed by the Feature-Model-Free system in response to CS and US presentation for each session of conditioning in the intermediate group. Simulated group is defined as in Figure 5. (TIFF) [file pcbi.1003466.s006.tiff]

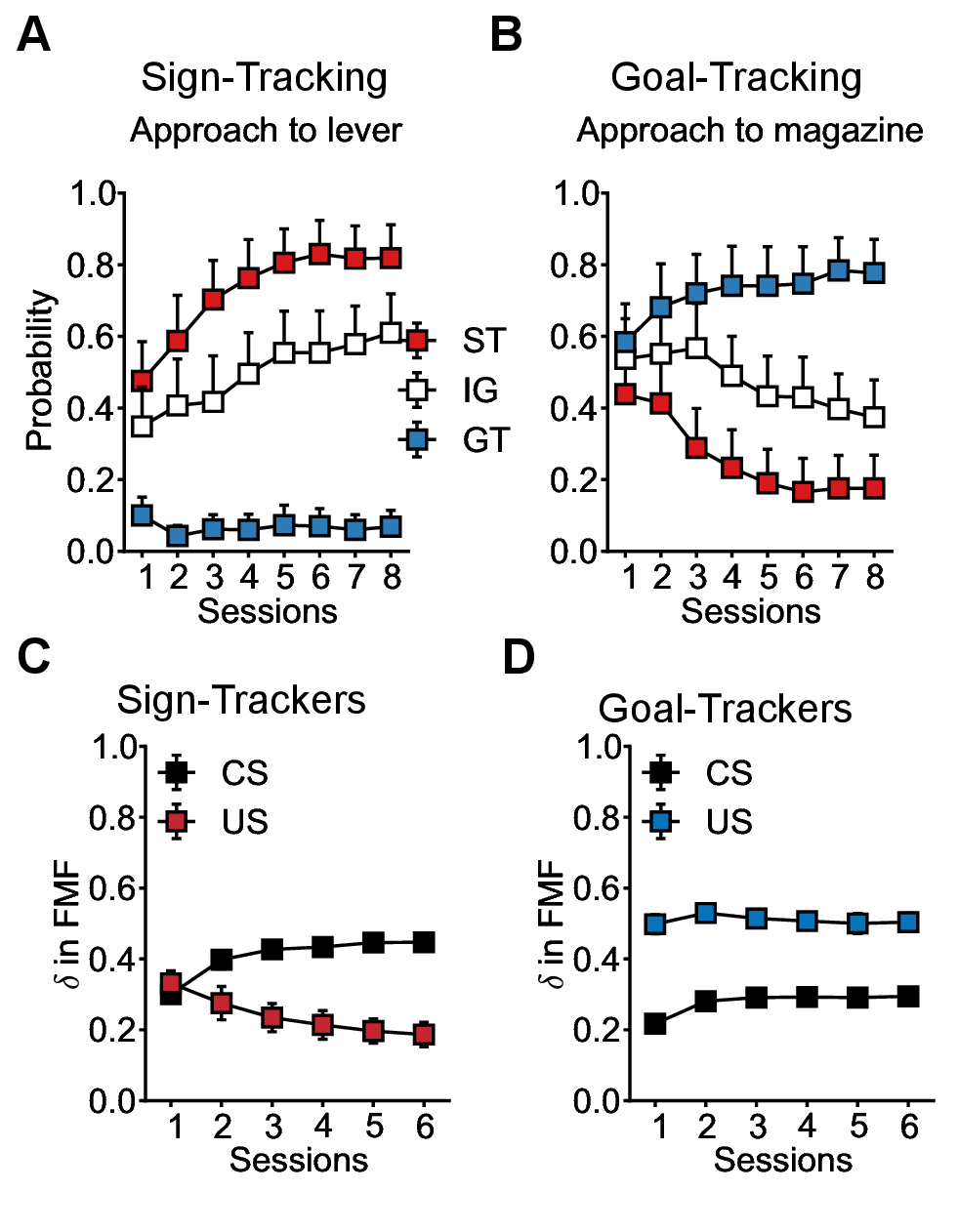

Supplement: Figure S7 — Behavioural and physiological simulations of autoshaping with shared parameter values across STs, GTs and IGs. (A,B) Legend is as in Figure 5 (C,D). Reproduction of the respective tendencies to sign- and goal-track of STs (), IGs () and GTs () using a single set of parameters (, , , , , and ). (C,D) Legend is as in Figure 7 (C,D). Reproduction of the different patterns of phasic dopaminergic activity in STs and GTs using the same single set of parameters. By simply varying the parameter, the model can still qualitatively reproduce the observations in experimental data. (TIFF) [file pcbi.1003466.s007.tiff]

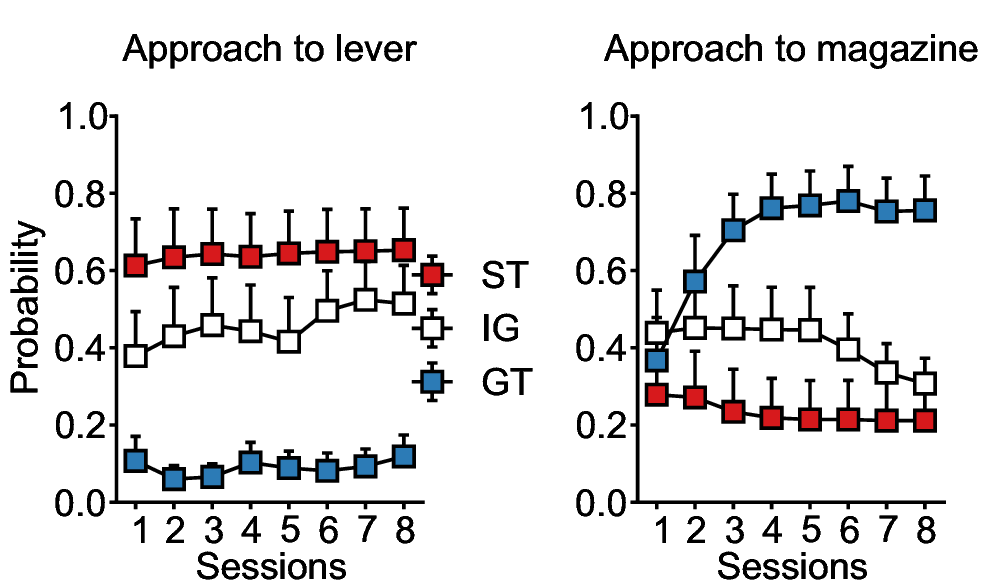

Supplement: Figure S8 — Simulation of autoshaping experiment for Variant 4. Legend is as in Figure 5 (C,D). Simulation for parameters STs (red), GTs (blue) and IGs (white) in the Variant 4 are summarized in Table S1. Variant 4 is not even able to reproduce the main behavioural data. (TIFF) [file pcbi.1003466.s008.tiff]

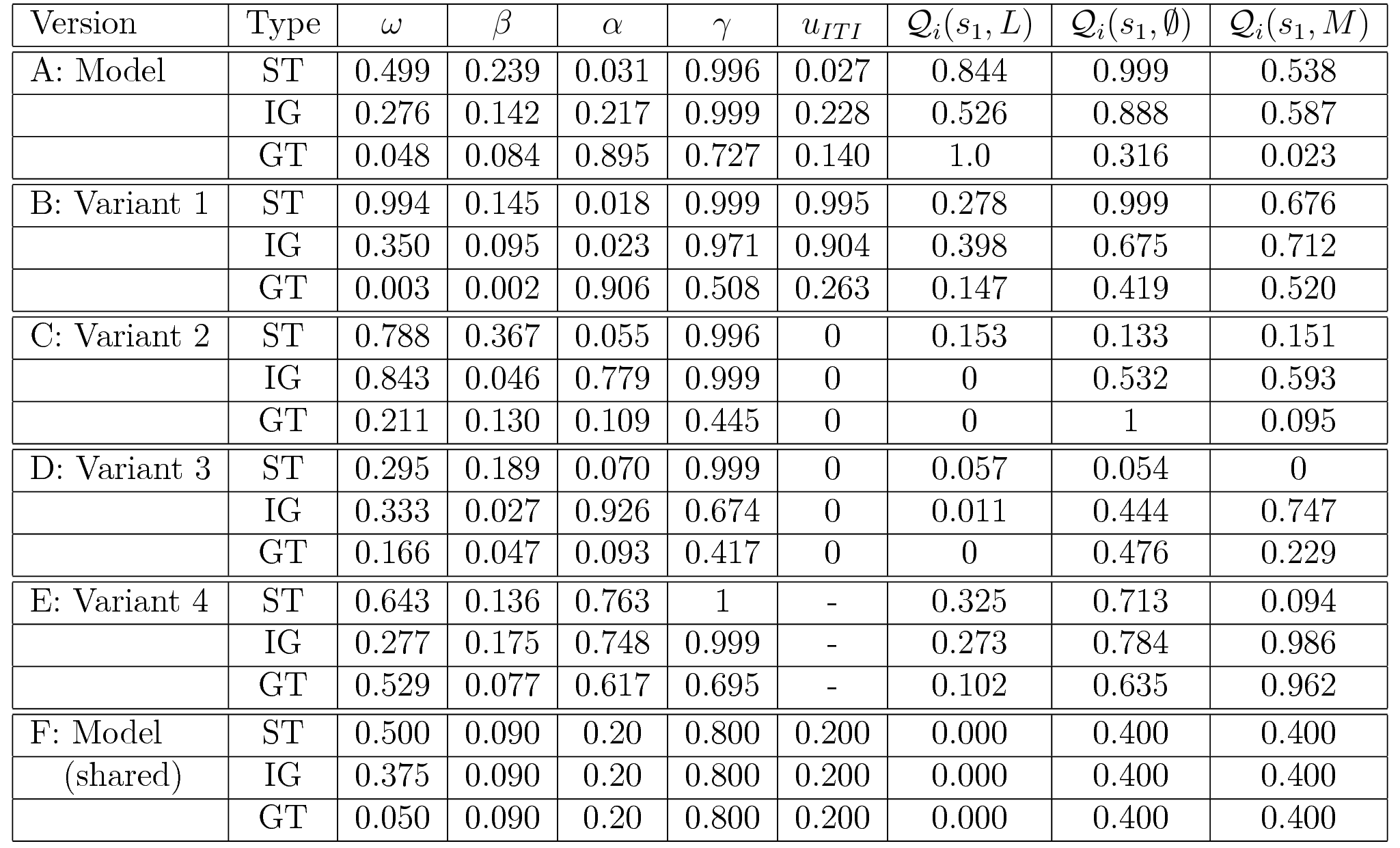

Supplement: Table S1 — Summary of parameters used in simulations. Parameters retrieved by optimisation with NSGA-II and used to produce the results presented in this article for the model and its variants. Parameters for STs, GTs and IGs were optimized separately (A,B,C,D,E). To confirm that is the key parameter of the model, we also optimized parameters for STs, GTs and IGs by sharing all but the parameter (F) to produce Figure S7. (TIFF) [file pcbi.1003466.s009.tiff]

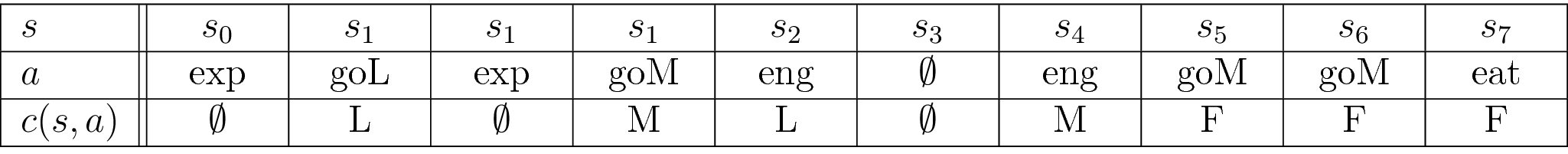

Supplement: Table S2 — Definition of feature-function . Stimuli (Lever, Magazine, Food or ) returned by the feature-function for each possible state-action pair in the MDP described in Figure 1. The feature-function simply defines the stimulus that is the focus of an action in a particular state. (TIFF) [file pcbi.1003466.s010.tiff]
